# A New Approach to Nipple-Sparing Mastectomy for Patients with Ptotic Breasts: A Single-Center, Retrospective Analysis of Feasibility and Treatment Outcomes

**DOI:** 10.3390/jcm15208026

**Published:** 2026-10-16

**Authors:** Paweł Pyka, Andrzej Frejnagel, Dzmitry Karkotka, Olga Nowak

**Affiliations:** 1 Clinical Department of Surgery, Wroclaw Medical University Branch Walbrzych, 50-367 Wałbrzych, Poland; 2 Department of Surgical Oncology, Alfred Sokolowski’s Specialistic Hospital in Walbrzych, 58-309 Wałbrzych, Poland; andfrej@wp.pl (A.F.); dmitry.karkotko@gmail.com (D.K.); 3 Student Research Group SKN, Wroclaw Medical University, 50-367 Wrocław, Poland; o.nowak@student.umw.edu.pl

**Keywords:** breast reconstruction, nipple-sparring mastectomy, skin-reducing mastectomy, breast cancer, breast implant

## Abstract

**Background/Objectives**: Subcutaneous mastectomies with immediate implant-based reconstruction have become widely performed procedures worldwide. Patients with small, non-ptotic breasts are candidates for procedures that do not involve skin reduction (NSM), whereas those with macromastia and significant ptosis undergo procedures involving the excision of excess skin (SRM). However, there remains a small group of patients for whom neither of these two options is ideal. We have developed a technique for reducing the implant pocket without excising excess skin, specifically designed for this group of patients. **Methods**: We conducted a retrospective analysis of 298 patients (419 breasts) who underwent surgery at our center between 2016 and 2025, comparing the method we described (NSM-LR) with NSM and SRM. A statistical analysis was performed. We evaluated oncological outcomes, complication rates, and implant loss. Satisfaction with the reconstructed breast was assessed using the Breast-Q questionnaire. **Results**: All cases were R0 resection. Locoregional recurrence was as follows: NSM—3.1%, NSM-LR—9.1%, and SRM—5.4% (*p* = 0.2363). Major complications were as follows: NSM—25.4%, NSM-LR—21.2%, and SRM—33.8% (*p* = 0.0887). Implant loss was as follows: NSM—7.6%, NSM-LR—10%, and SRM—17.6% (*p* = 0.0155). Breast Q score was as follows: NSM—78, NSM-LR—82, and SRM–75. **Conclusions**: NSM-LR can be a reasonable option for selected patients requiring a subcutaneous mastectomy.

## Simple Summary

Subcutaneous mastectomies with immediate reconstruction have become an essential part of every breast surgeon’s skill set. There are two main surgical techniques used for subcutaneous mastectomies with implant-based reconstruction: one without skin reduction—for patients with small, non-ptotic breasts—and a reduction technique involving the excision of excess skin for patients with large, ptotic breasts. Most female patients can easily be assigned to one of them. However, there remains a small group of patients for whom neither of these two options is ideal. We present a retrospective analysis of our own technique, which allows for limited breast reduction without skin excision, compared to techniques involving skin reduction and those without it. We found no significant differences in oncological treatment outcomes. Major complications and implant loss were higher in the group undergoing excess skin removal. Patient satisfaction with the reconstructed breasts was comparable. In summary, we can recommend our technique as an option for selected patients requiring subcutaneous mastectomy.

## 1. Introduction

**Figure 1:**
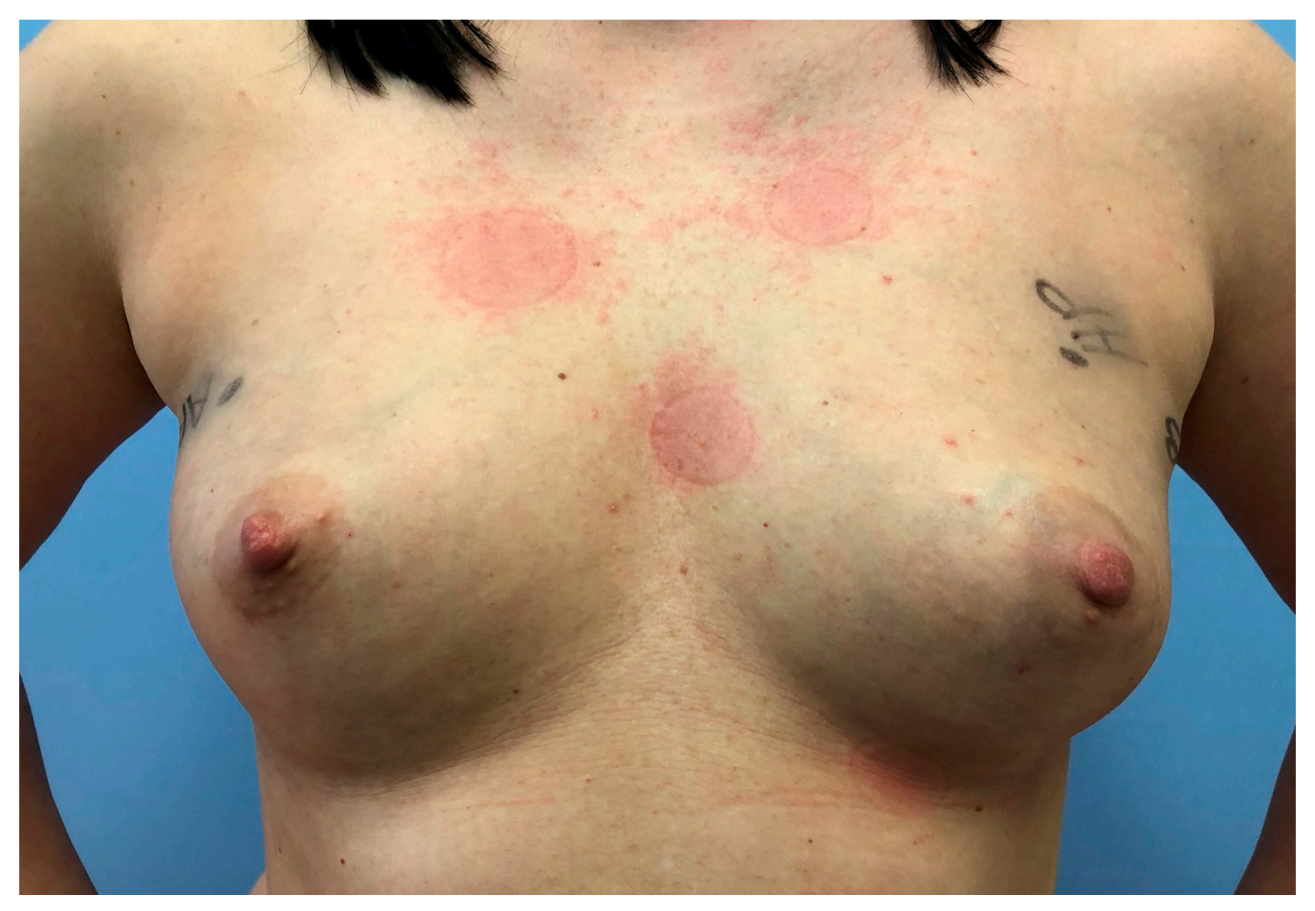
Before NSM.

**Figure 2:**
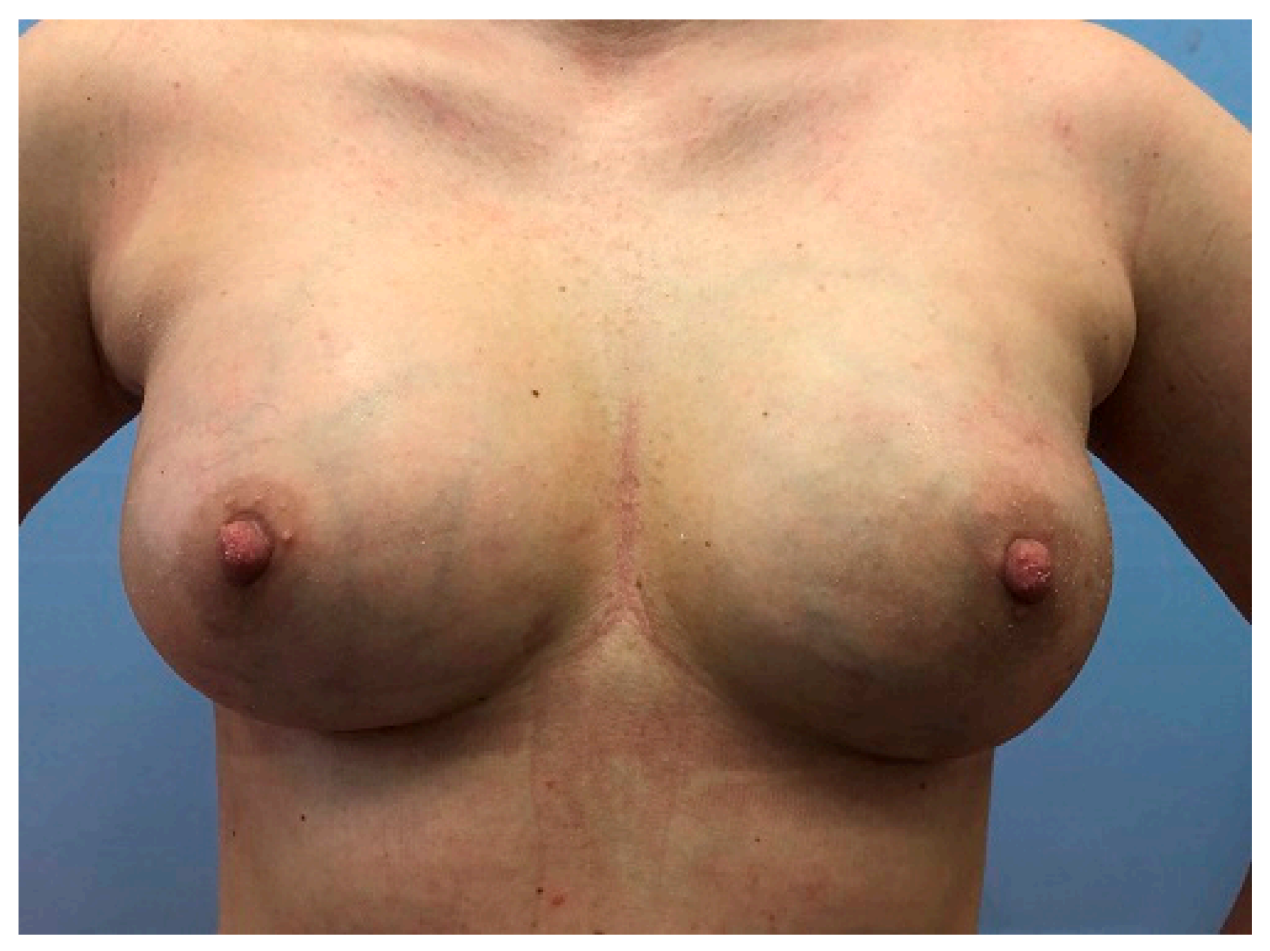
After NSM.

Breast cancer is the most common cancer among women worldwide [1]. The “gold standard” for the surgical treatment of breast cancer is breast-conserving surgery, which involves removing the tumor while maintaining margins free of cancer cells [2]. Mastectomy remains an alternative treatment for breast cancer when breast-conserving surgery is not possible [3,4]. However, the loss of a breast has a profound impact on body image, as well as psychosocial and sexual well-being [5,6]. Consequently, breast reconstruction has become an integral part of comprehensive oncological treatment, rather than merely an aesthetic procedure. Therefore, in every case where there are no contraindications, the patient should be offered a subcutaneous mastectomy with immediate reconstruction. Implant-based reconstructions are the most commonly used methods for immediate procedures [7]. Despite the evident benefits, these procedures carry a risk of complications, the most serious of which include ischemia of the skin flaps, impaired wound healing, and infections [8,9]. Properly selecting the patient for the appropriate surgical technique plays a key role in reducing the number of complications. The risk of reconstruction failure increases with the mass/volume of the implant used [9,10]. Similarly, extensive skin excision generates a higher risk of ischemia and implant exposure [9,11,12]. There is no doubt that patients with small- to medium-sized breasts and without excessive ptosis are ideal candidates for subcutaneous mastectomy without skin envelope reduction, namely nipple-sparing mastectomy (NSM) (Figure 1 and Figure 2).

**Figure 3:**
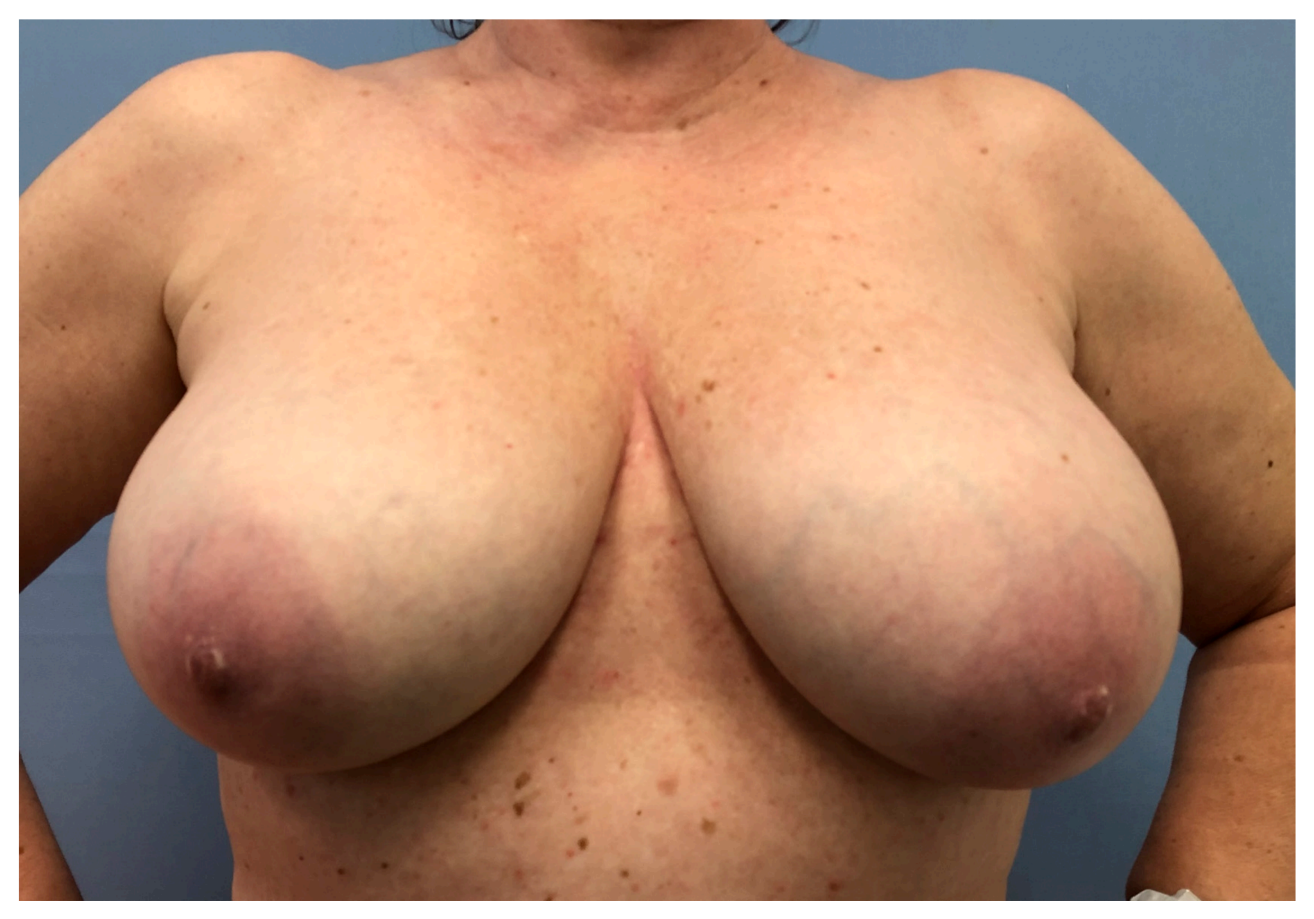
Before SRM.

**Figure 4:**
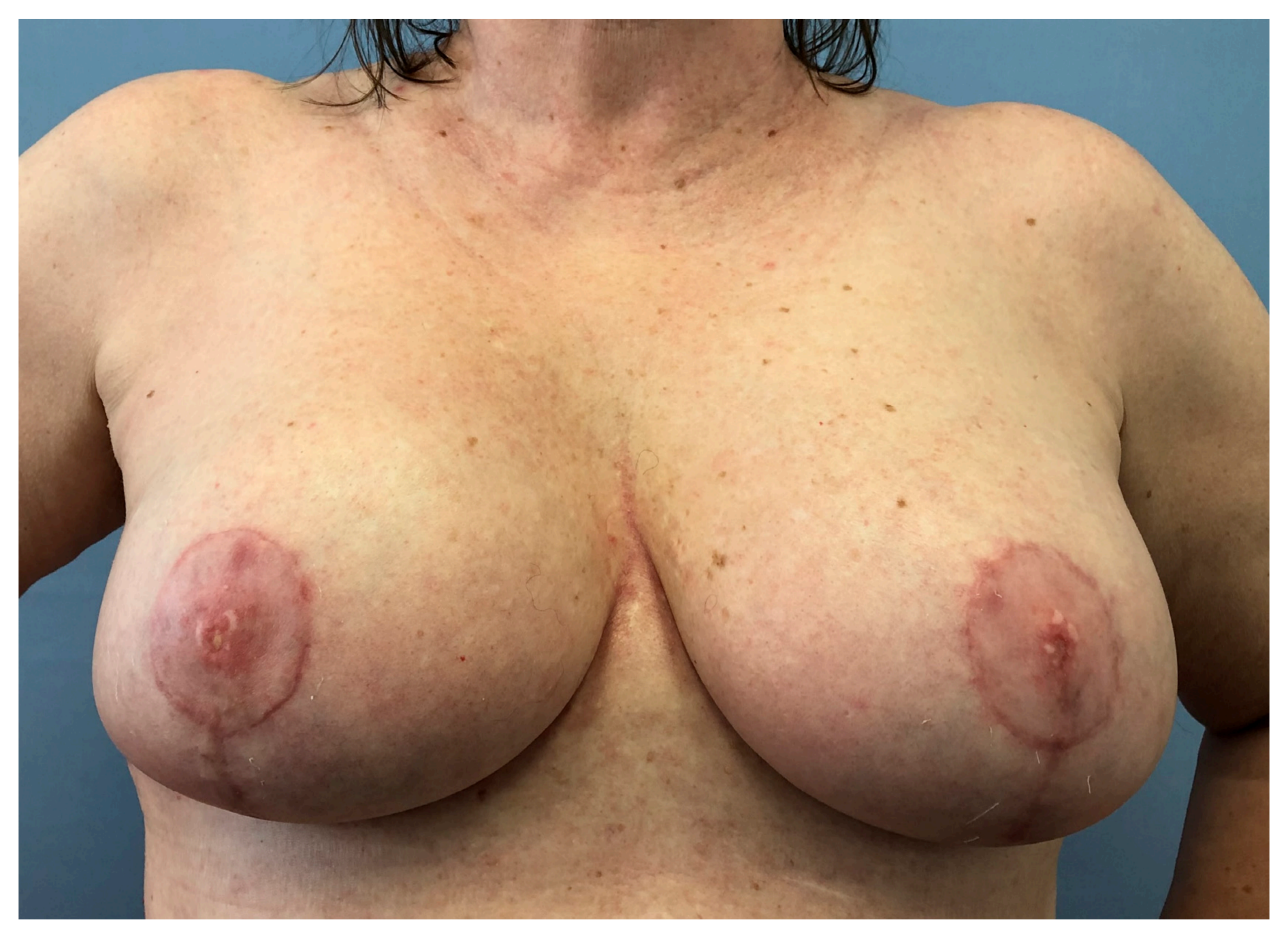
After SRM.

For patients with macromastia and/or significantly ptotic breasts, the procedure of choice is one that includes skin reduction, skin-reducing mastectomy (SRM) (Figure 3 and Figure 4).

**Figure 5:**
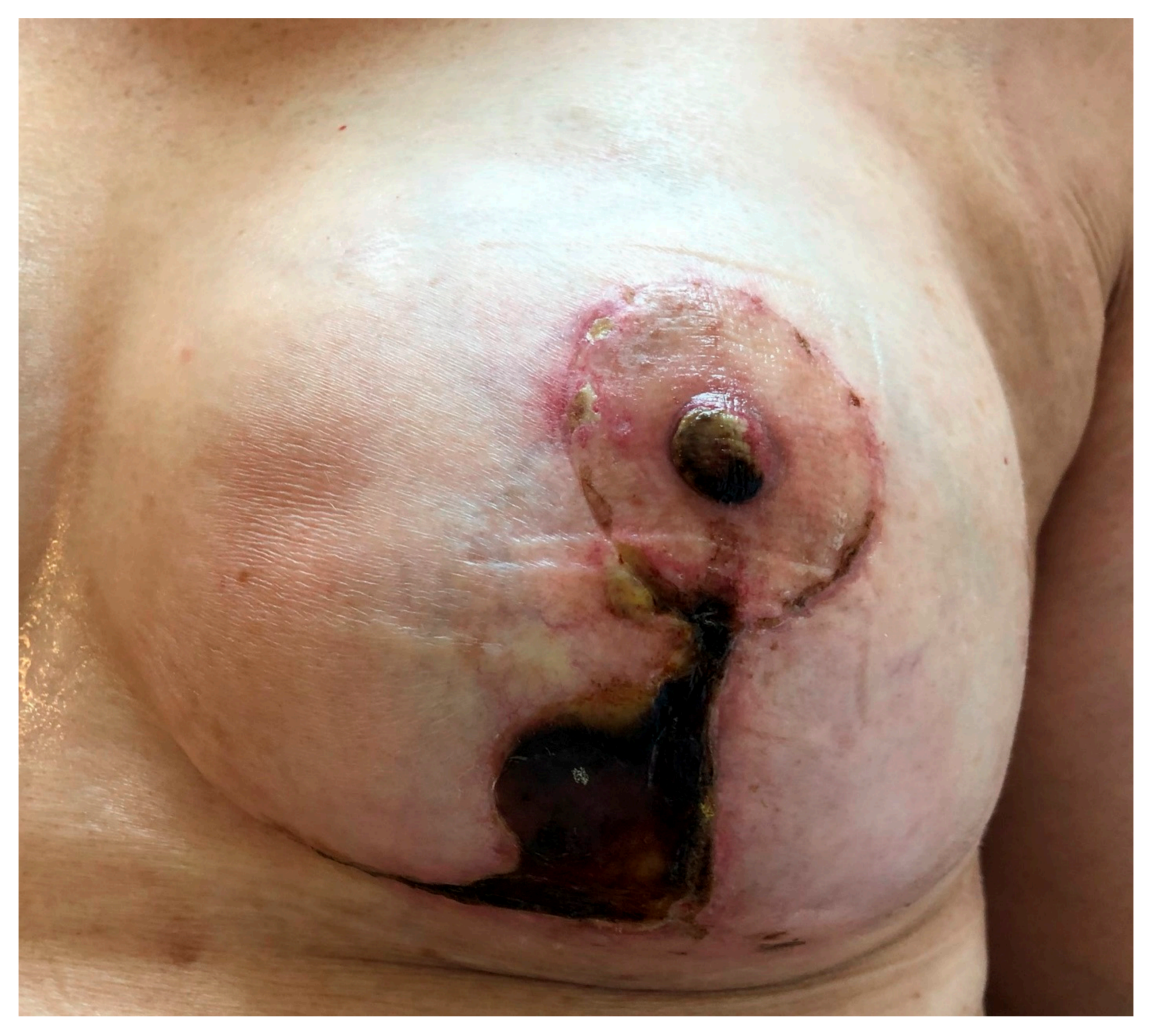
Wise pattern necrosis.

**Figure 6:**
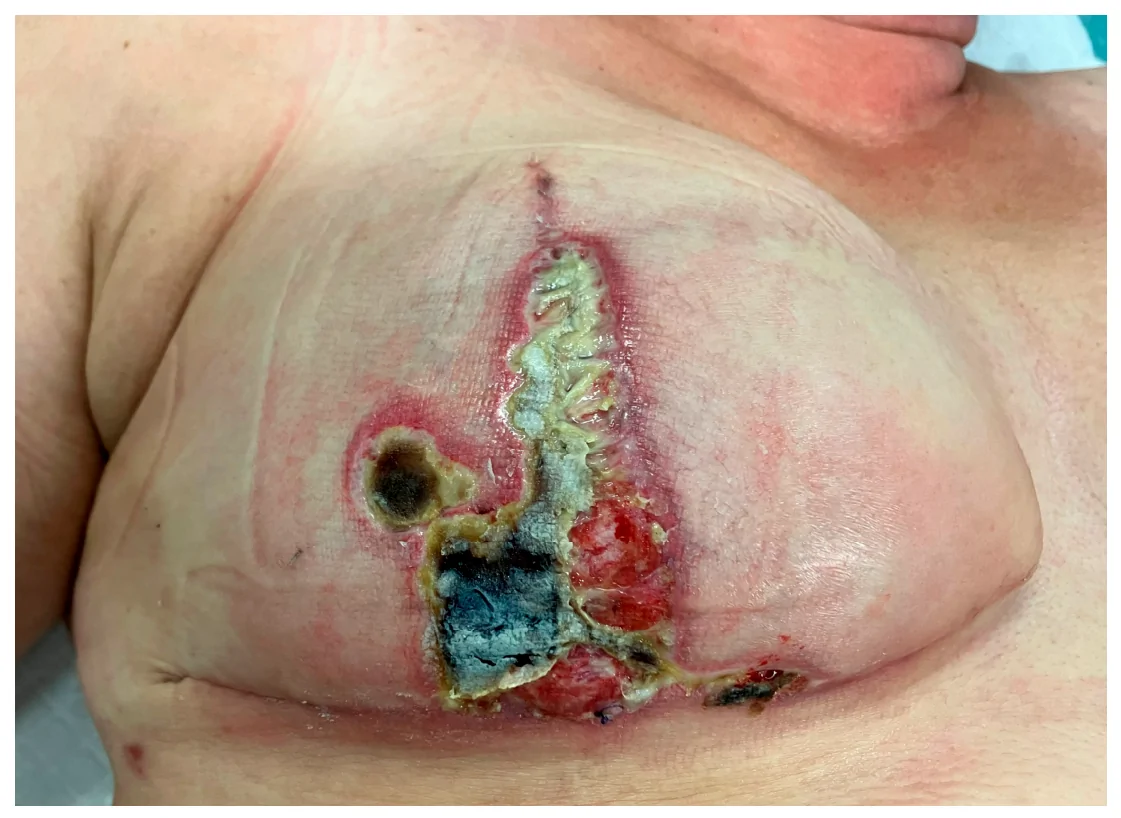
Wise pattern necrosis.

However, there is a group of patients that falls between these two types. Their breasts are not particularly large and ptosis is not significant. Determining the suitability of such patients for surgery presents a challenge. Choosing a procedure that does not involve skin reduction would require the use of a large implant to correct breast ptosis. However, not all patients desire such a solution. Moreover, a larger and heavier implant may increase the risk of complications [13]. An alternative is to perform a procedure involving the excision of excess skin to reduce the size of the pocket in which the implant will be placed. Unfortunately, this also carries the risk of an increased number of complications. Postoperative wound necrosis at the junction of the skin flaps leads to a high rate of complications, resulting in implant loss and reconstruction failure [14,15]. There is no doubt that it is associated with an extensive full-thickness skin incision that compromises the blood supply to the remaining skin flaps [16]. This is clearly evident in the course of the necrosis located at the edge of the vertical incision used in the “Wise pattern” technique (Figure 5 and Figure 6).

Patients with moderate ptosis and without severe macromastia undoubtedly require a reduction in the skin envelope but do not need excision of the excess skin.

Since 2018, our center has employed a technique that allows for the reduction in the skin envelope without excising the excess skin. It also eliminates the vertical cut line. It should avoid the adverse effects of excess skin excision and reduce the number of associated complications. For the purposes of this paper, we have named this technique NSM-LR (Nipple-Sparing Mastectomy with Limited Reduction).

The aim of this study is to present this surgical technique and evaluate its effectiveness, as well as demonstrating a reduction in the number of complications compared to skin-reducing mastectomy.

## 2. Materials and Methods

### 2.1. Study Design and Participants

We conducted a single-center, retrospective analysis of patients operated on in the Department of Surgical Oncology of the Dr. Alfred Sokolowski Specialist Hospital in Walbrzych. The database comprised consecutive patients who, between 2016 and 2025, underwent subcutaneous mastectomy with immediate reconstruction as a part of breast cancer treatment or a risk-reducing/prophylactic procedure. We decided to analyze each breast separately because, in several cases, the two breasts of a single patient were operated on using different techniques. The initial database included 309 patients and 436 breasts.

**Figure 7:**
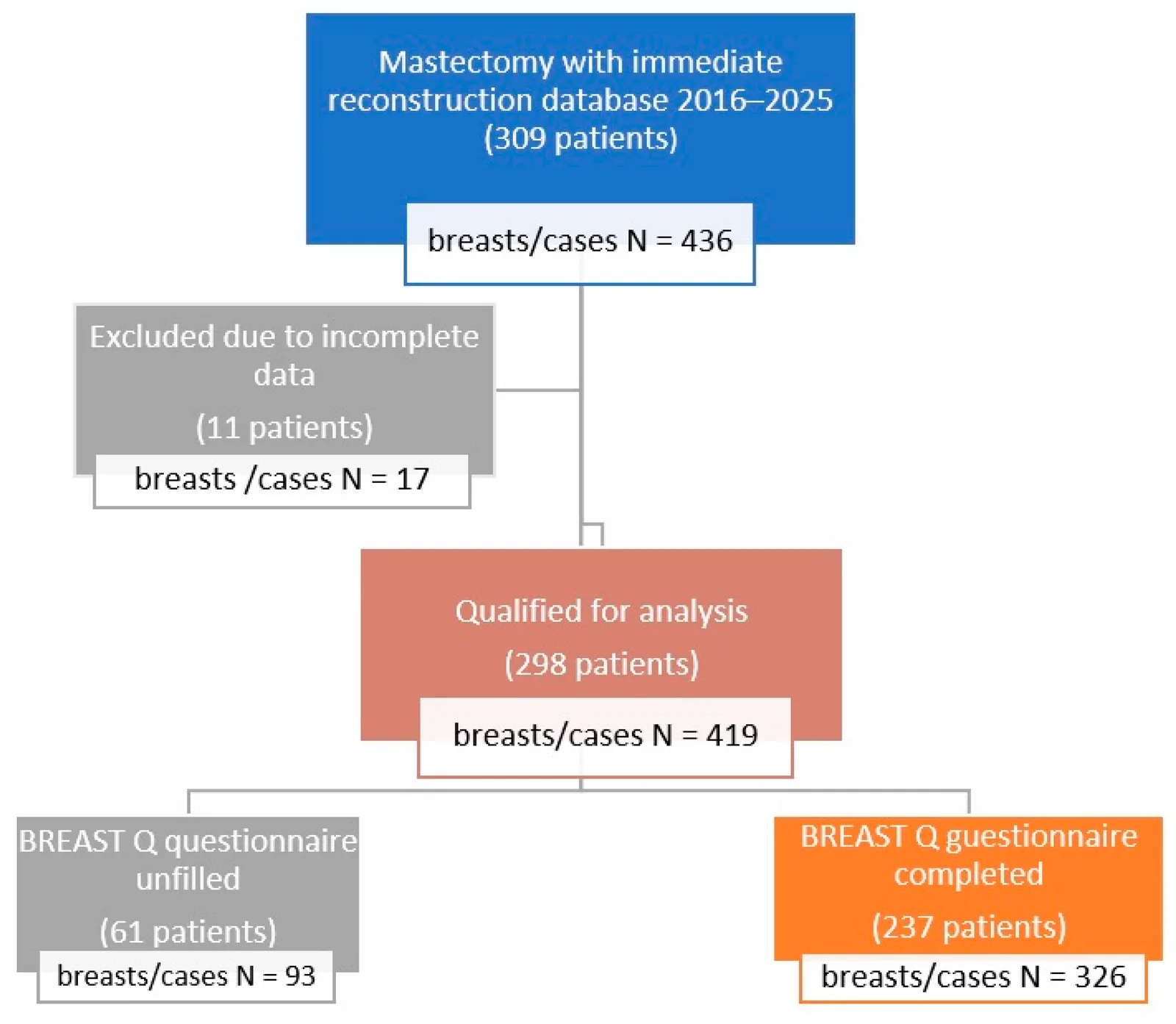
Patient selection for the analysis. From the initial database of 436 cases, incomplete data were excluded, yielding a cohort of 419 post-mastectomy cases. Breast Q questionnaire-completed subgroup included 326 cases.

Eligibility criteria for the final analysis were: (1) mastectomy with immediate implant-based reconstruction and (2) access to the complete medical history, enabling assessment of the postoperative course and the management of complications. In accordance with the predefined analysis plan, records with incomplete data were excluded. Ultimately, 298 patients met the criteria and 419 breasts were analyzed. Not all patients answered the questions included in the BREAST-Q questionnaire; therefore, the cohort used to assess satisfaction with the surgery is smaller than the entire analyzed group. Ultimately, it includes 237 patients and 326 breasts (Figure 7).

**Figure 8:**
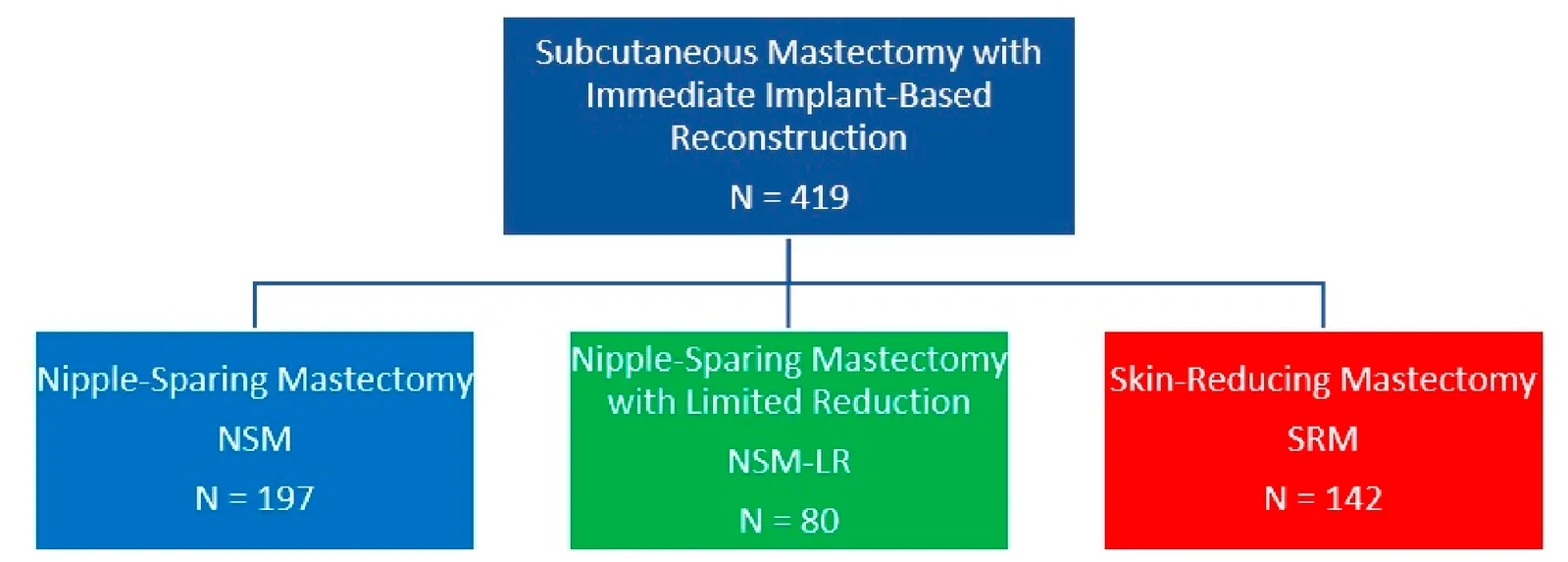
Cases division into three analyzed groups: NSM, NSM-LR, and SRM.

The cases selected for analysis were divided into three groups based on the surgical technique employed: NSM (nipple-sparing mastectomy without any skin reduction), NSM-LR (nipple-sparing mastectomy with limited reduction but without skin excision), and SRM (skin-reducing mastectomy with excision of excess skin) (Figure 8).

### 2.2. Description of the Surgical Technique

#### 2.2.1. NSM-LR (Nipple-Sparing Mastectomy with Limited Reduction)

##### Surgical Procedure Planning

**Figure 9:**
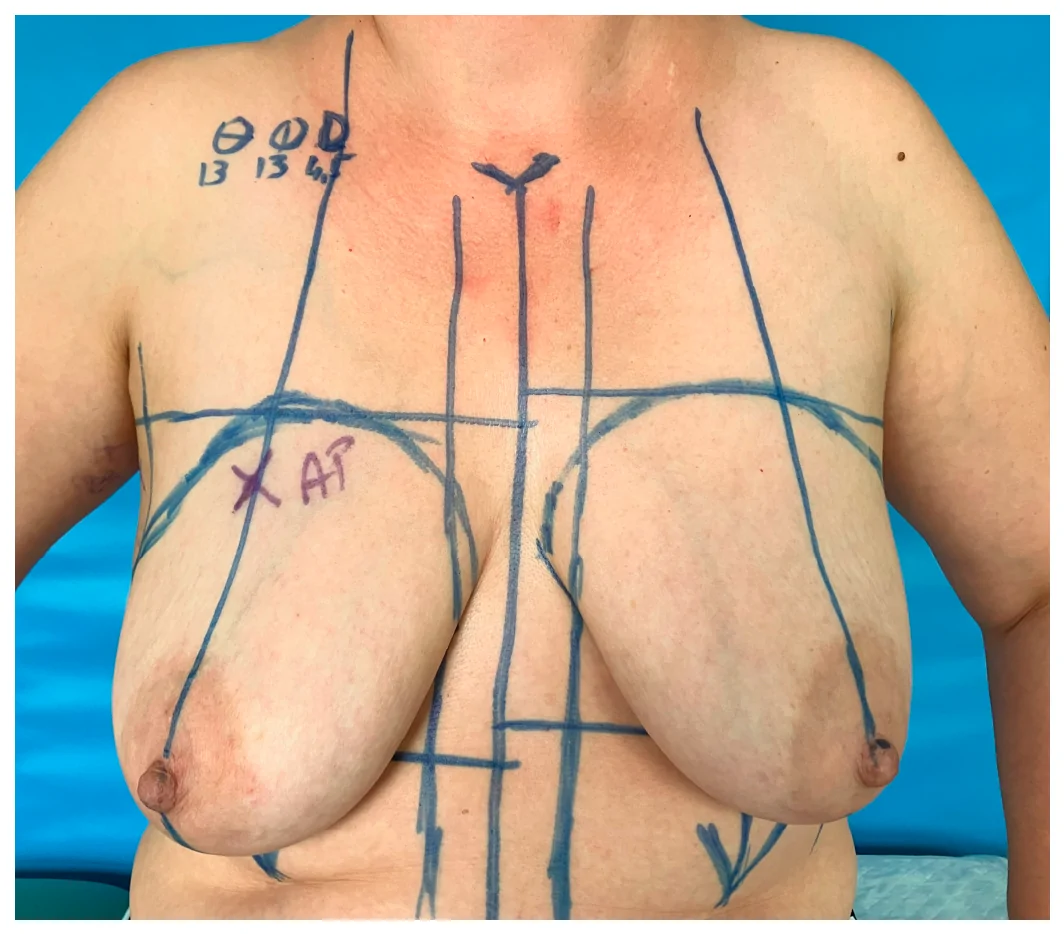
Typical NSM marking.

**Figure 10:**
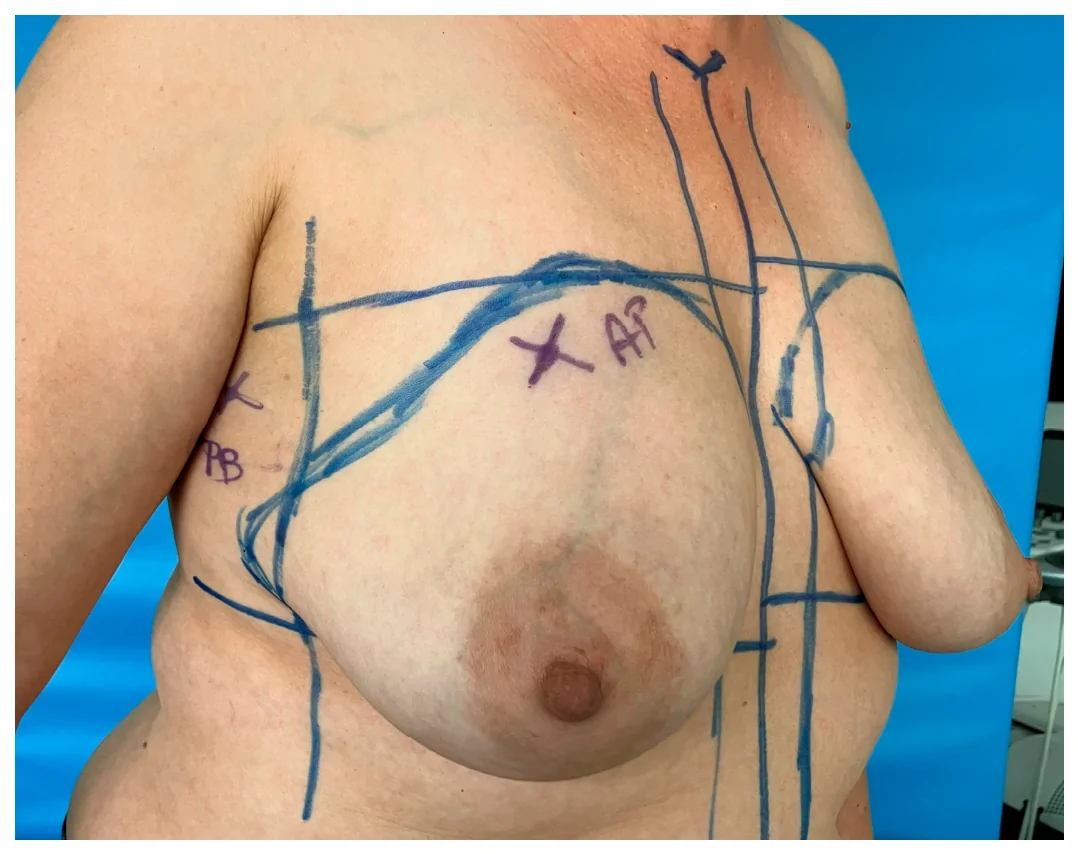
Typical NSM marking.

Surgical planning begins by marking the lines typical of a subcutaneous mastectomy: a line running down the center of the sternum; parasternal lines (which also serve as the medial margin for the implant); anterior axillary lines (defining the lateral limit of the implant); and lines connecting the midpoint of the clavicle to the nipple, representing the axis of the breast. The inframammary fold and the upper border of the mammary gland are also marked (Figure 9 and Figure 10).

At this stage, the implant’s width, height, and projection can be selected.

**Figure 11:**
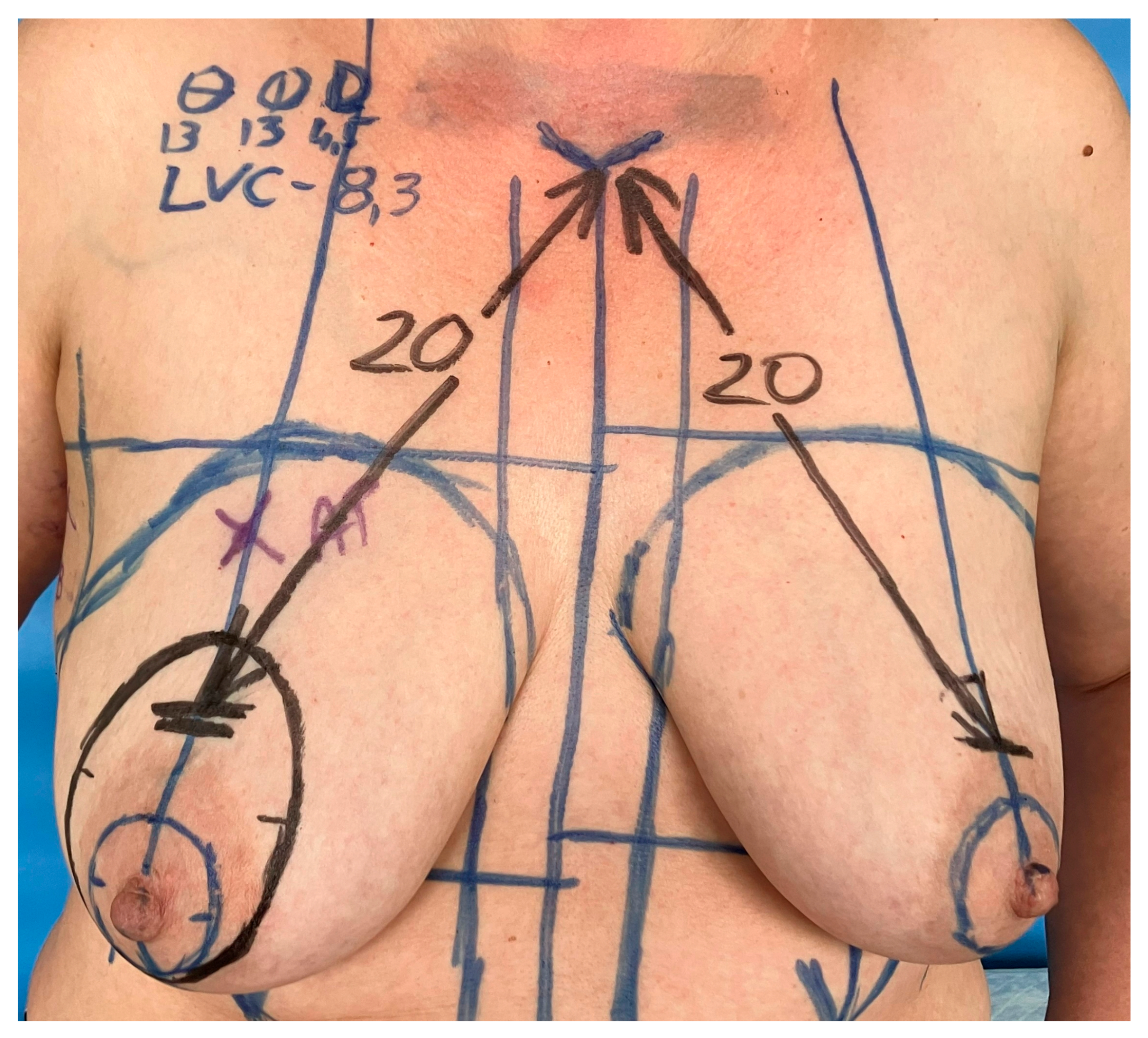
Future location of the nipple.

Next, the future position of the nipple is determined (there are many methods for determining nipple placement, and the specific method used does not matter for the technique described). Knowing the future nipple position and the planned diameter of the nipple–areola complex (NAC), the location of its upper margin can be determined (Figure 11).

**Figure 12:**
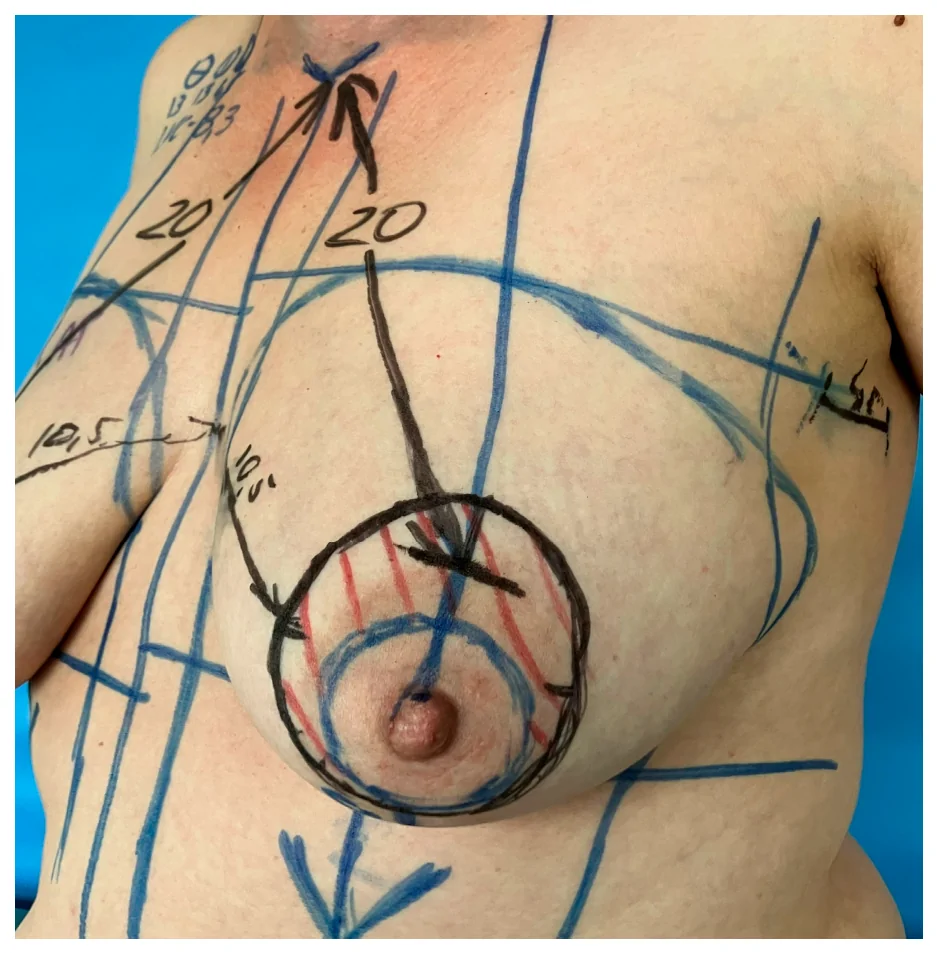
Outlined area around the NAC.

By connecting the upper margin of the planned NAC location to the lower margin of its current location with an oval line (encircling the NAC) and outlining the NAC at its planned diameter, we define the area that will be de-epithelialized during the procedure to allow for the NAC’s relocation (Figure 12).

**Figure 13:**
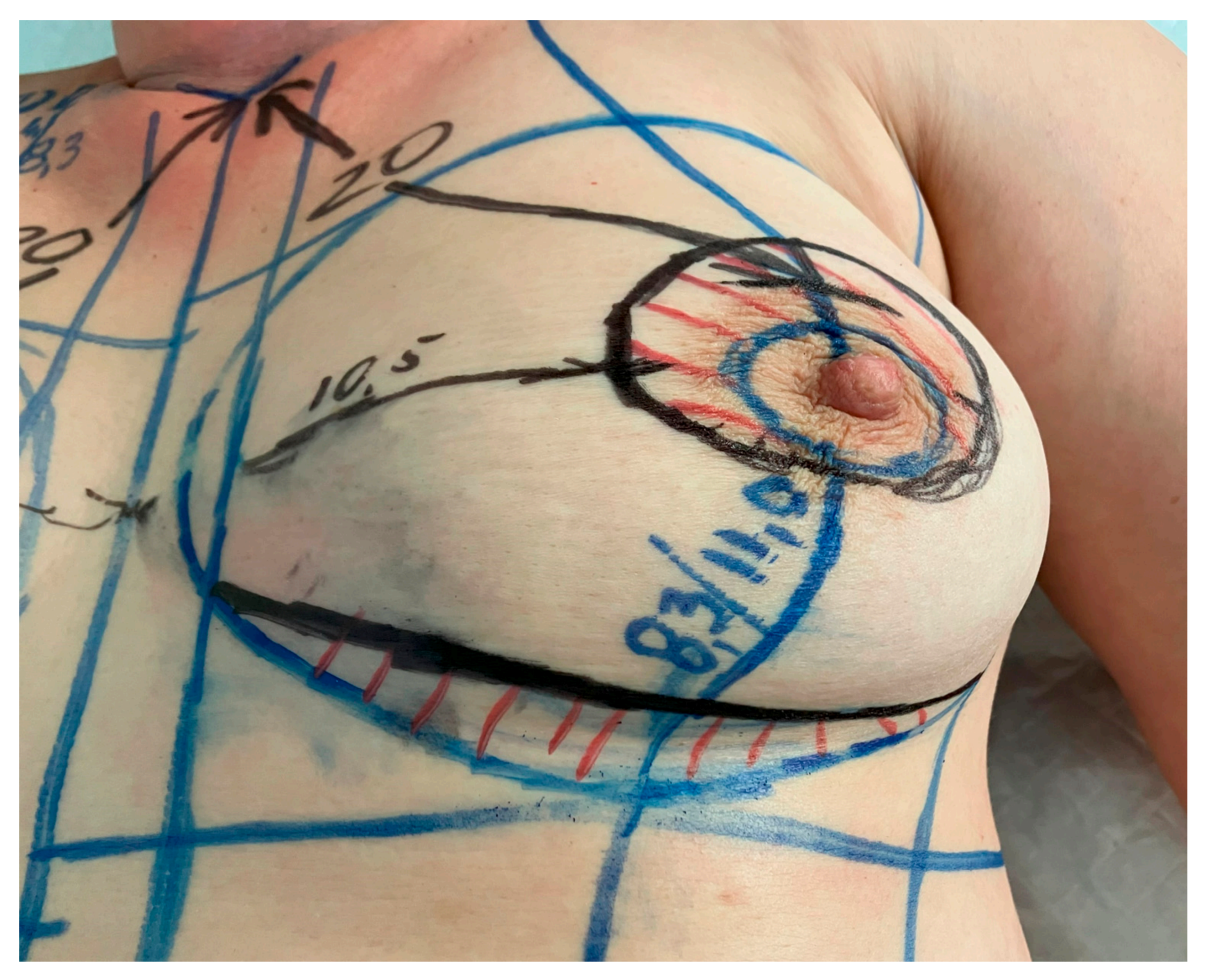
Previous and future inframammary fold.

When selecting the implant, we note the manufacturer-specified distance from its apex to its lower edge (LVC). This corresponds to the distance from the nipple to the inframammary fold. Knowing this dimension allows us to determine where the inframammary fold should be positioned on the reconstructed breast. We mark this distance by measuring from the nipple along a line extending the breast axis toward the inframammary fold. Then, by connecting the marked point to the parasternal and anterior axillary lines, we outline the future path of the inframammary fold. The area between the current and future inframammary fold will undergo de-epithelialization during the procedure to reduce the skin envelope (Figure 13).

**Figure 14:**
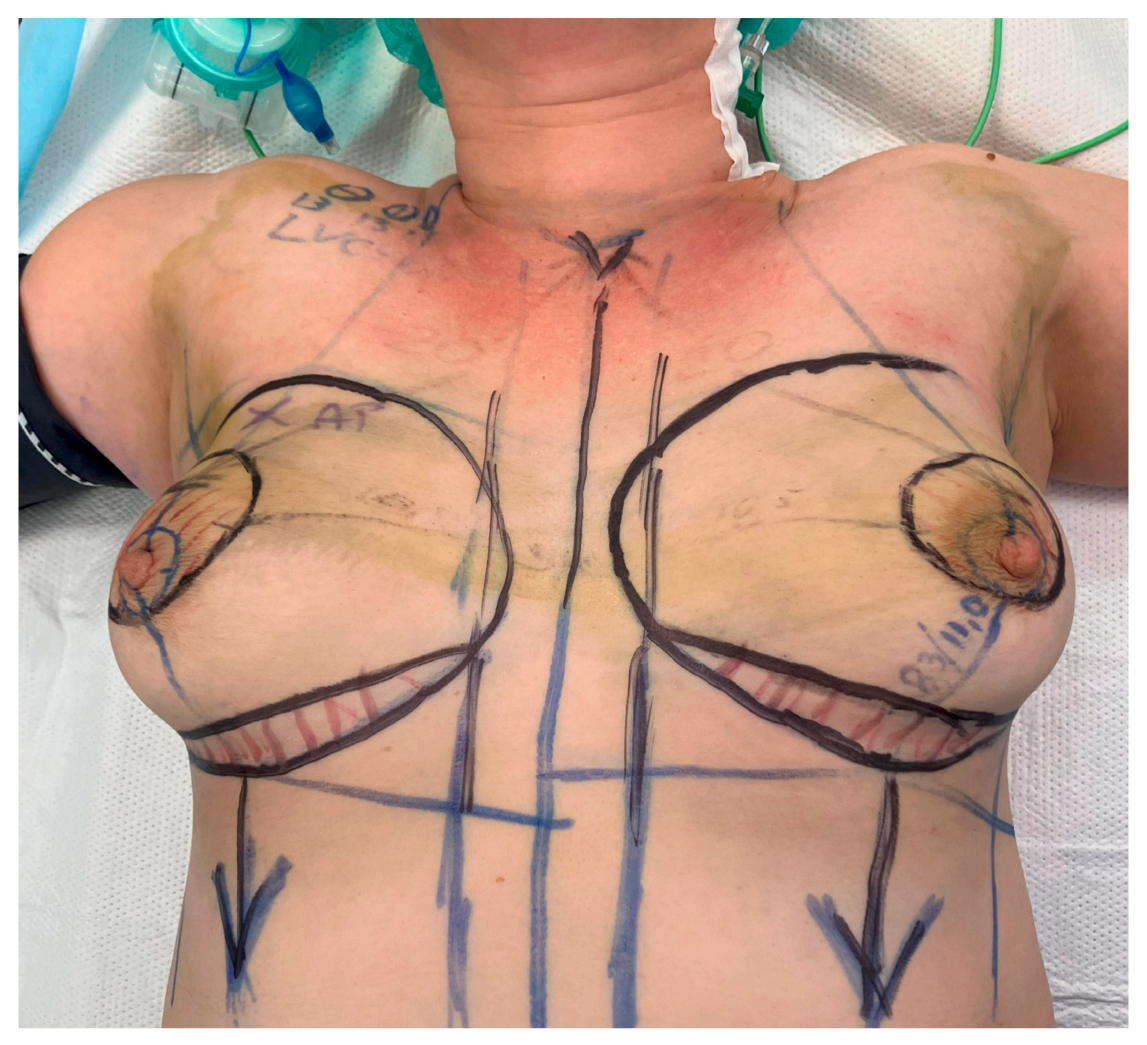
Planning completed.

At this stage, surgical planning is complete (Figure 14).

##### Performing Surgery

**Figure 15:**
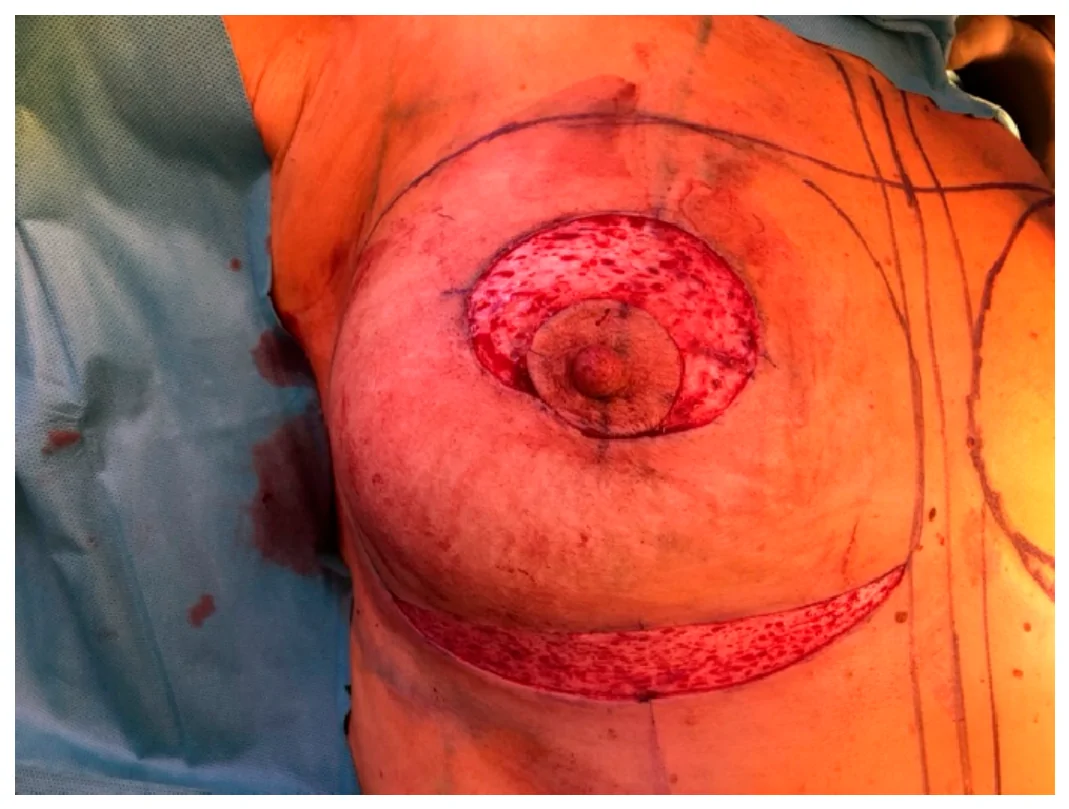
De-epithelialized areas.

**Figure 16:**
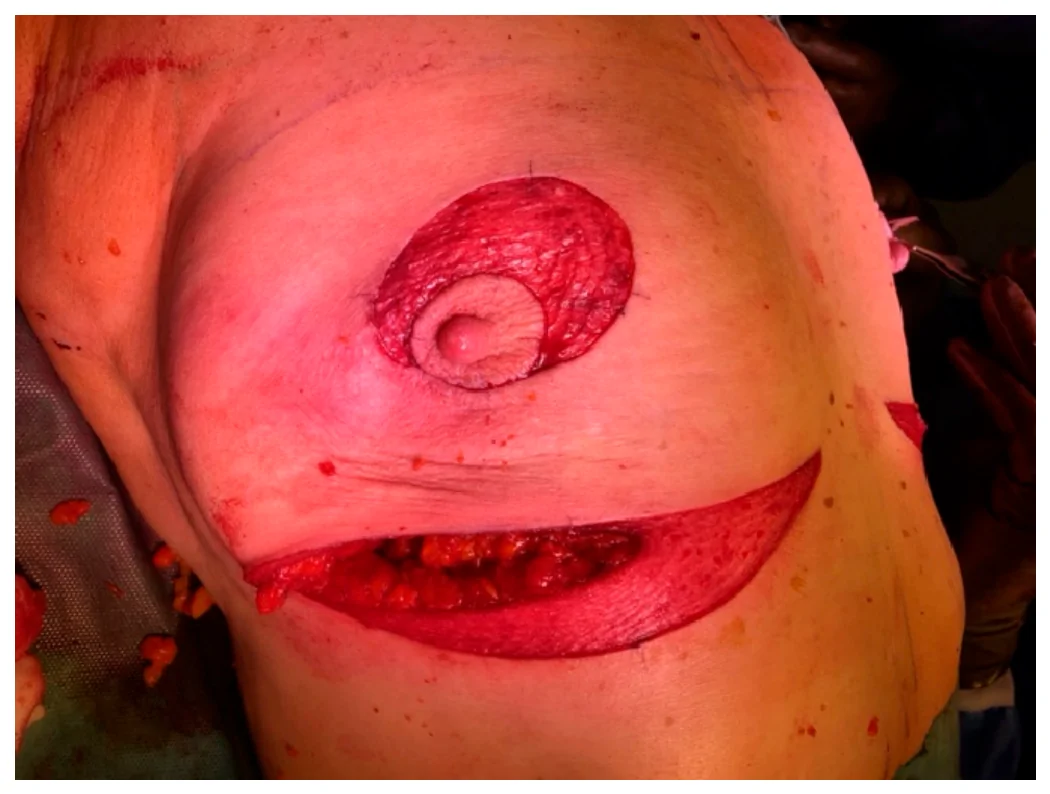
Skin incision in the inframammary fold.

The surgical procedure begins with de-epithelialization of the previously marked areas in the inframammary fold and around the NAC (Figure 15). Subcutaneous mastectomy is performed via an incision made in the lateral part of the de-epithelialized area within the inframammary fold (Figure 16).

We recommend not extending the incision beyond the breast midline to avoid compromising the skin’s blood supply—specifically the branches of the intercostal arteries running from the epigastrium toward the lower breast quadrants and their accompanying veins. Making the incision in this manner also preserves the small sensory nerve branches running from the nipple.

**Figure 17:**
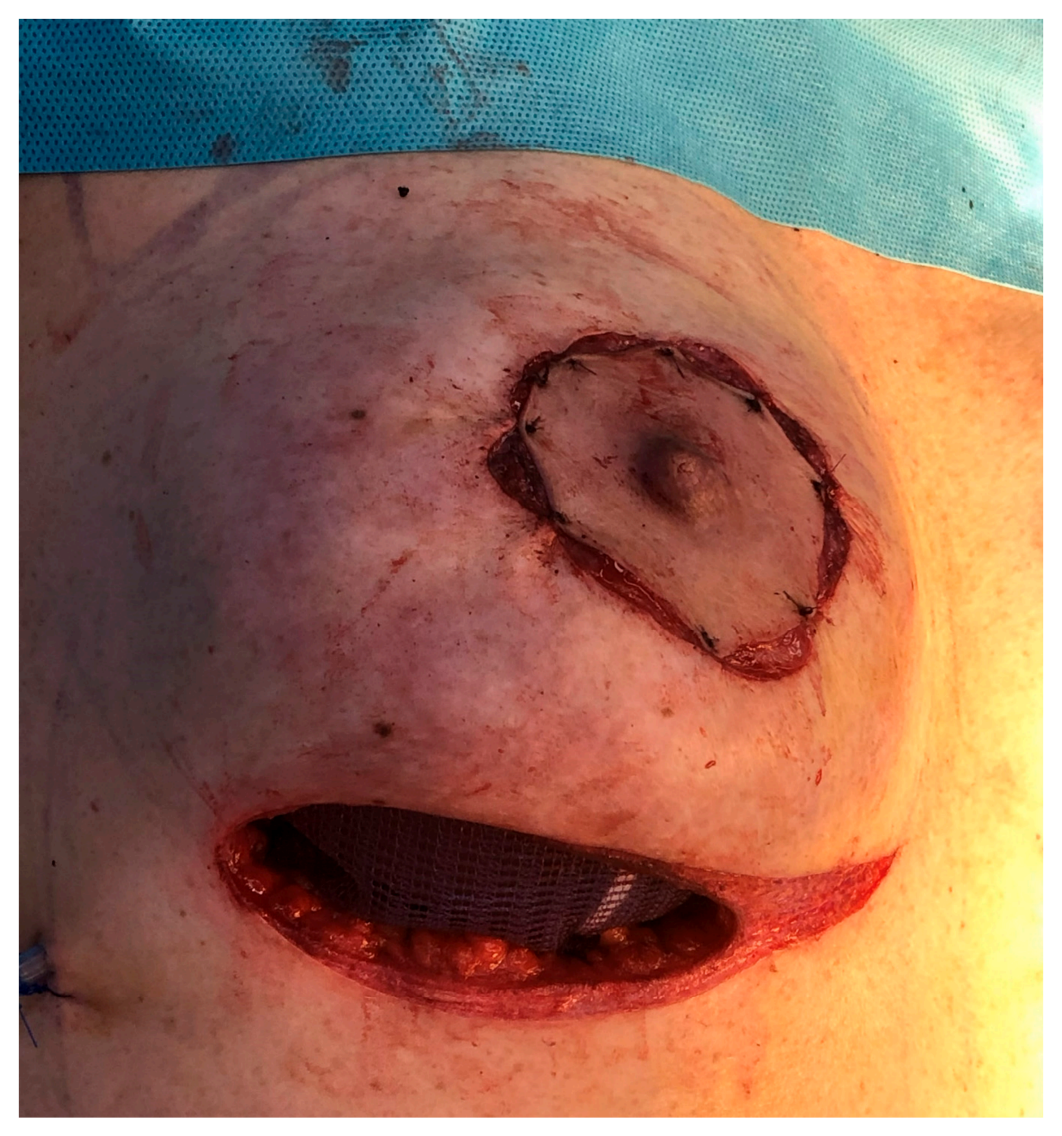
Wound closure.

**Figure 18:**
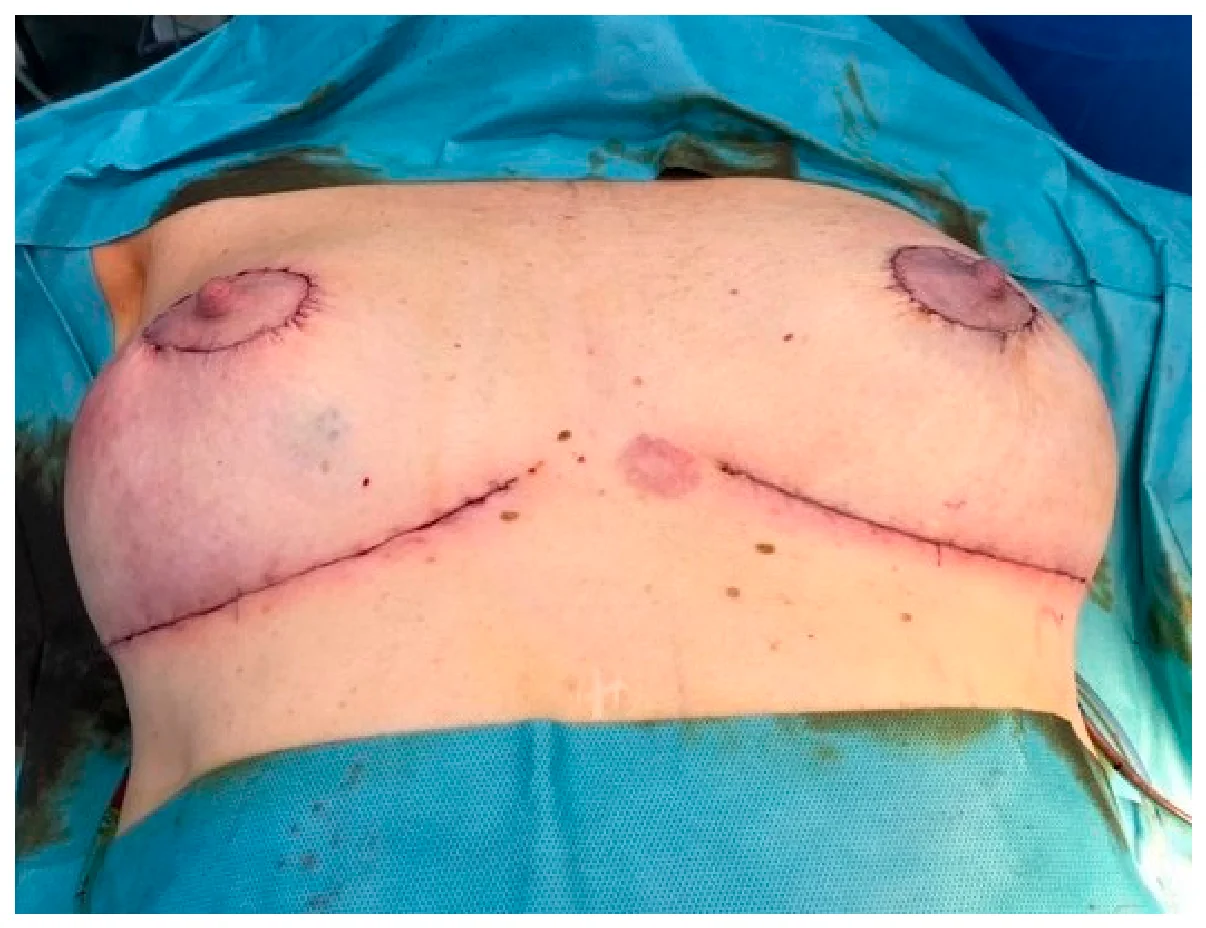
Surgery completed.

The gland is dissected at the plane bordering the subcutaneous tissue, in the same manner as for a nipple-sparing mastectomy. Following gland removal and hemostasis, the pre-selected breast implant is inserted through the same incision. The author of the technique advocates placing a drain in the implant pocket, though this is not mandatory. The procedure is finished by the layered closure of the de-epithelialized area in the inframammary fold and the region surrounding the nipple–areola complex (NAC) (Figure 17). This allows for the repositioning of the NAC to a central location on the reconstructed breast and the achievement of an adequate distance between the nipple and the inframammary fold (Figure 18).

**Figure 19:**
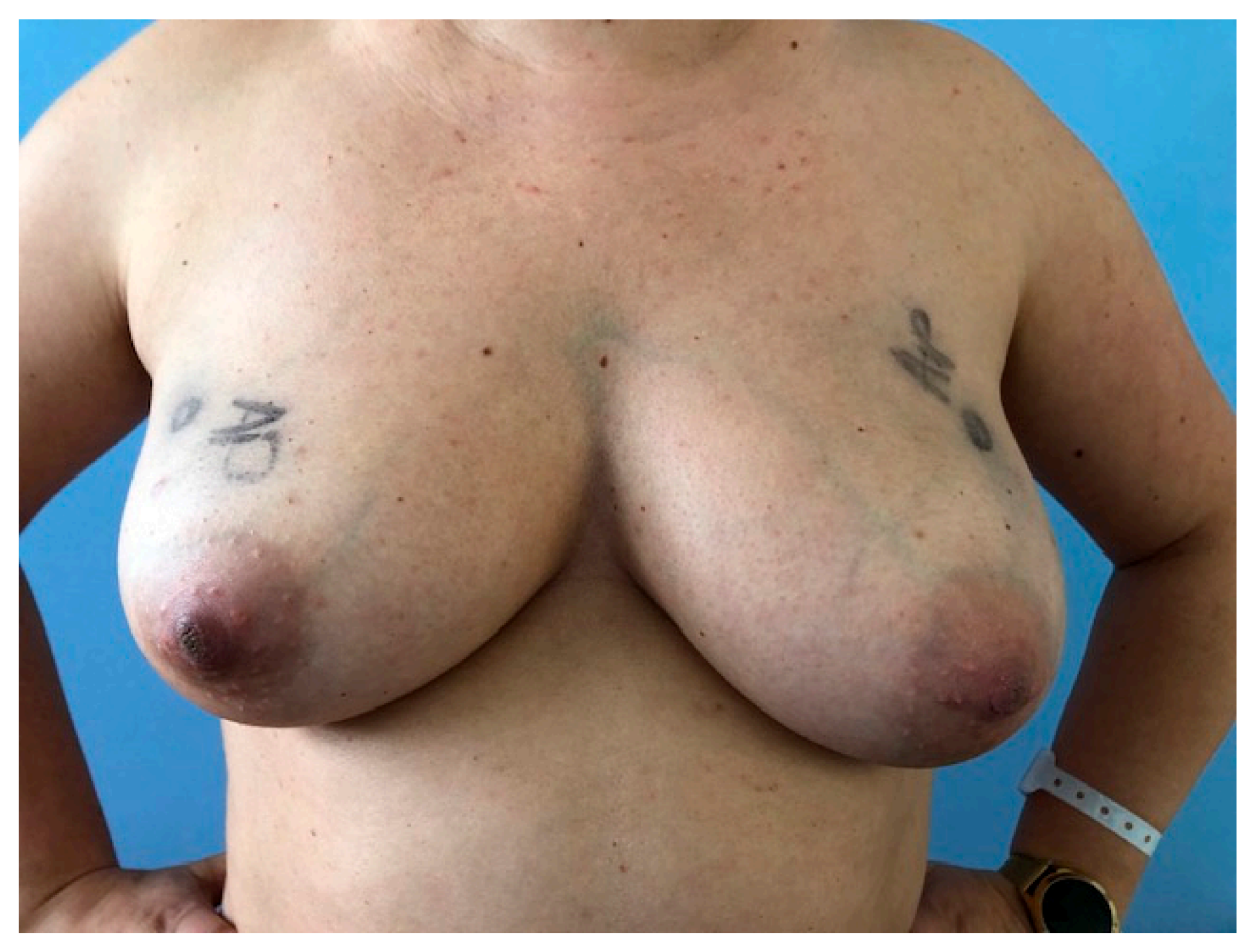
Before NSM-LR.

**Figure 20:**
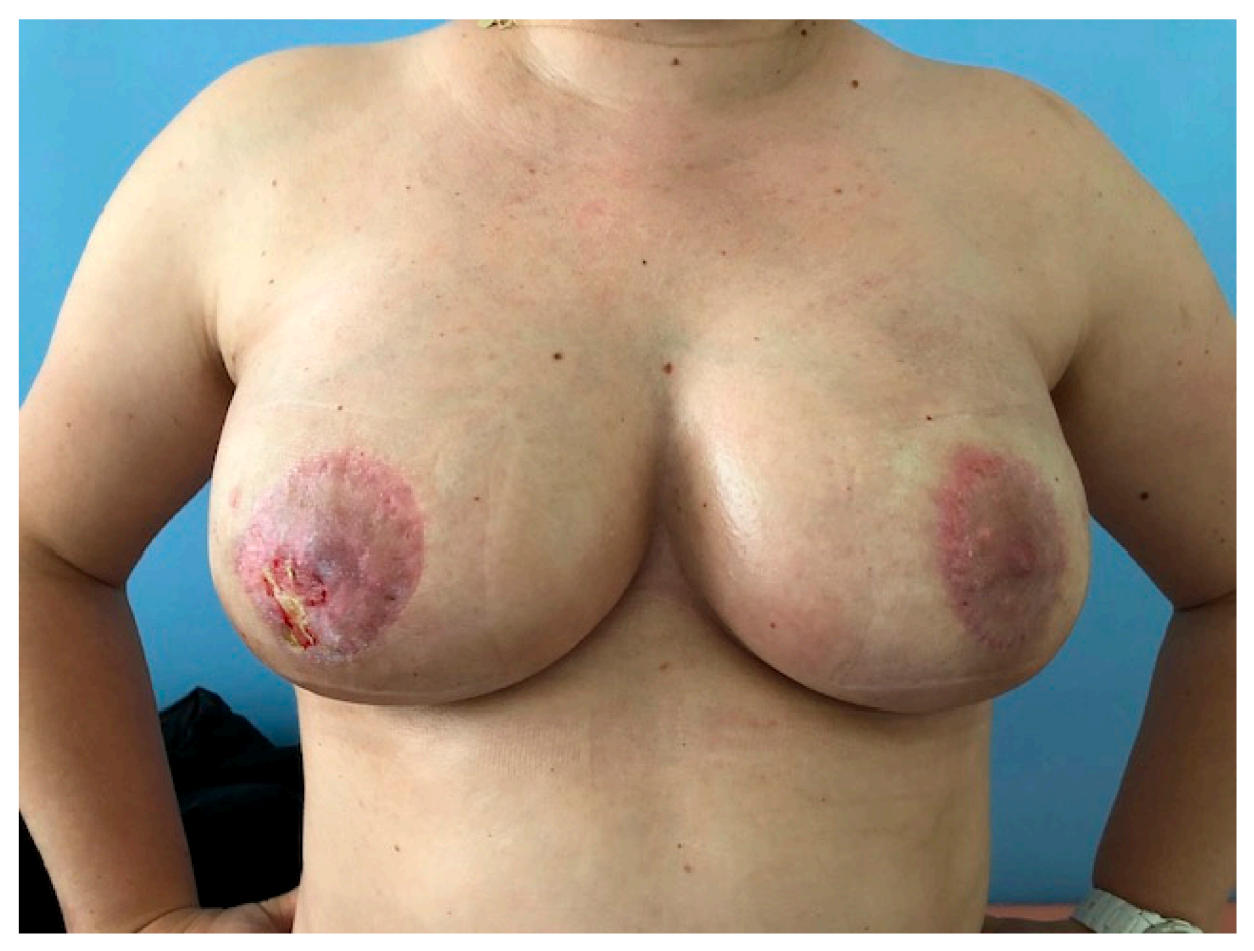
Four weeks after NSM-LR.

**Figure 21:**
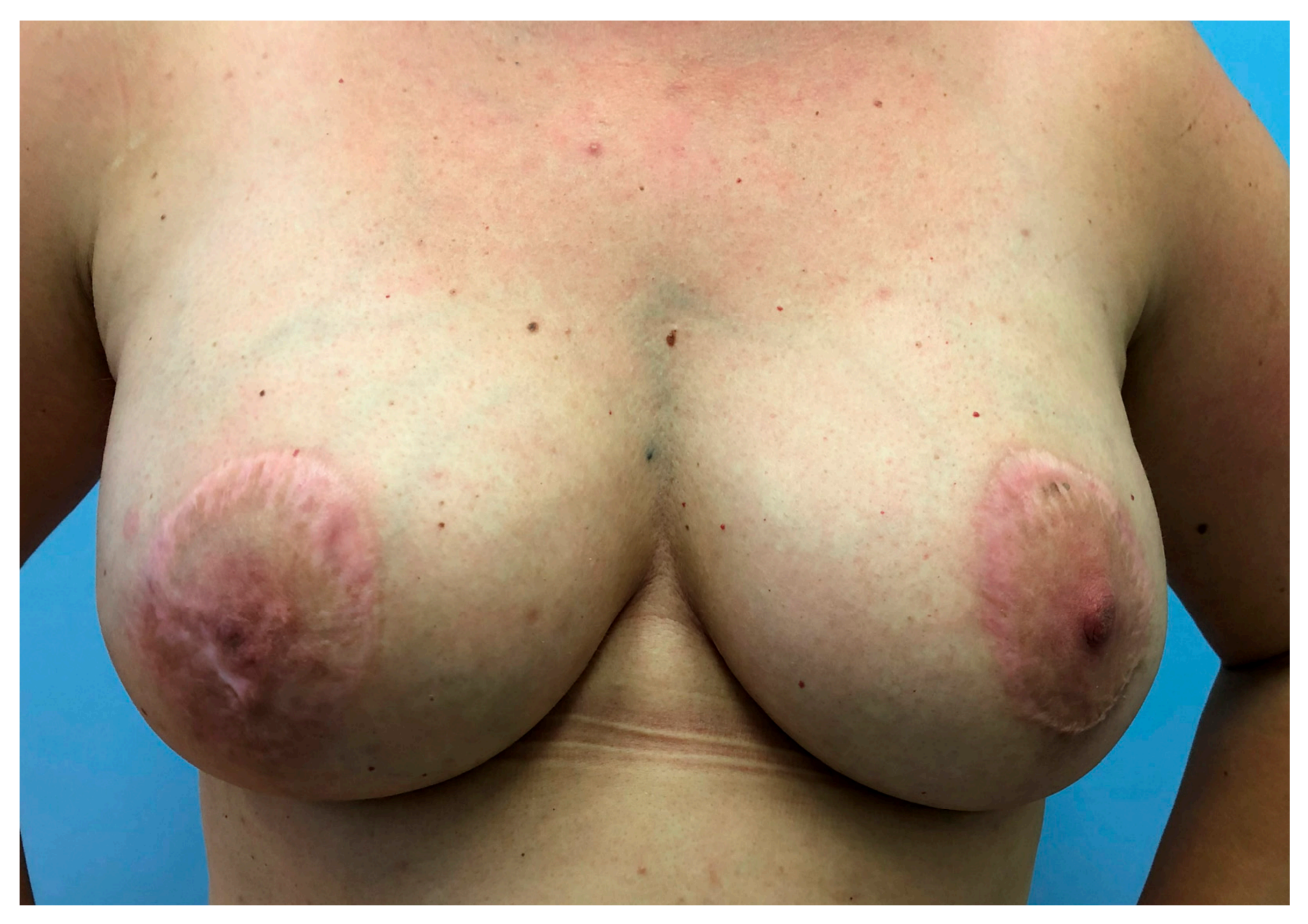
Six months after NSM-LR.

The following photographs show the results obtained four weeks and 6 months after surgery (Figure 19, Figure 20 and Figure 21). Superficial necrosis of the right NAC is visible; this is the most common complication following this procedure.

**Figure 22:**
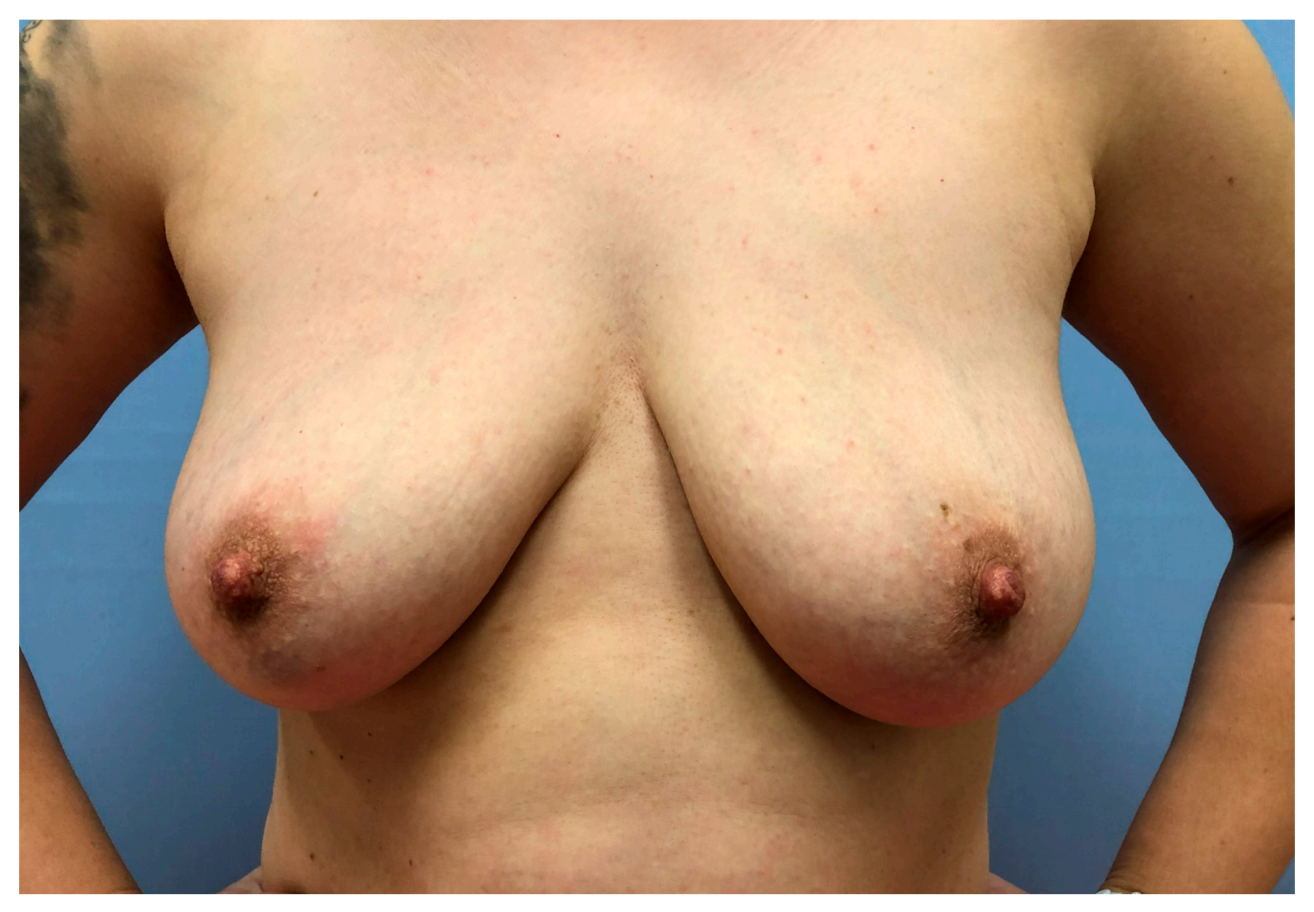
Before NSM-LR.

**Figure 23:**
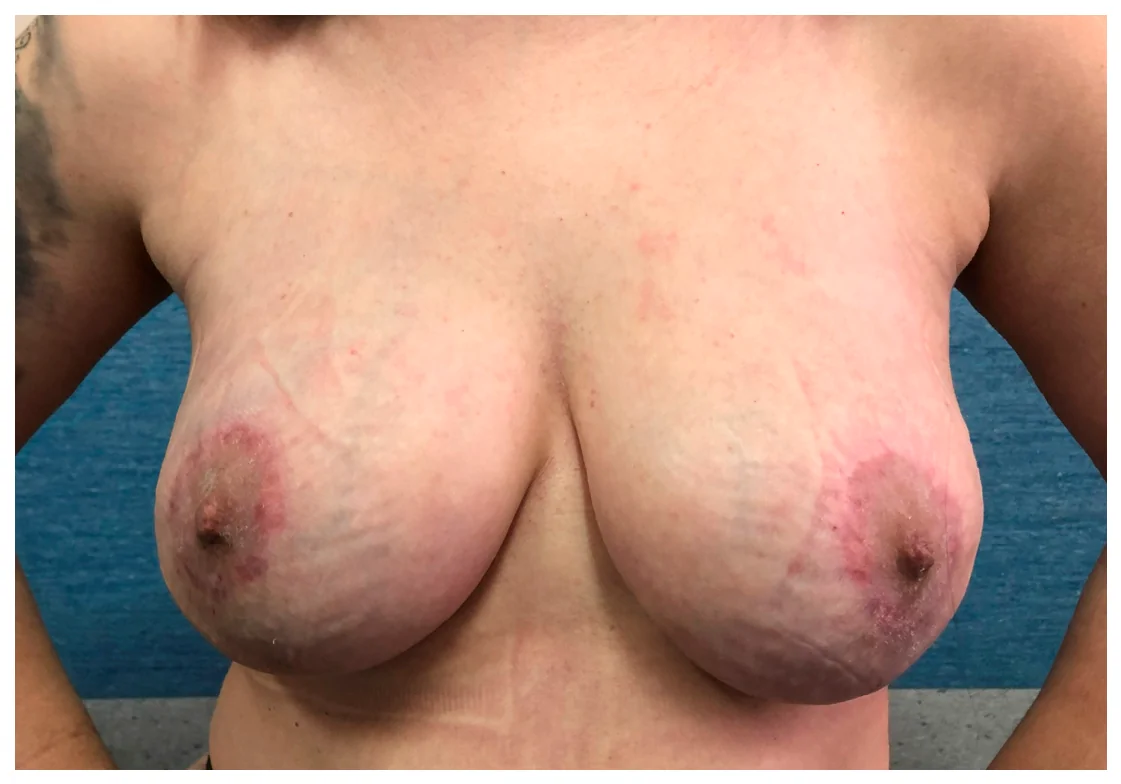
Six months after NSM-LR.

**Figure 24:**
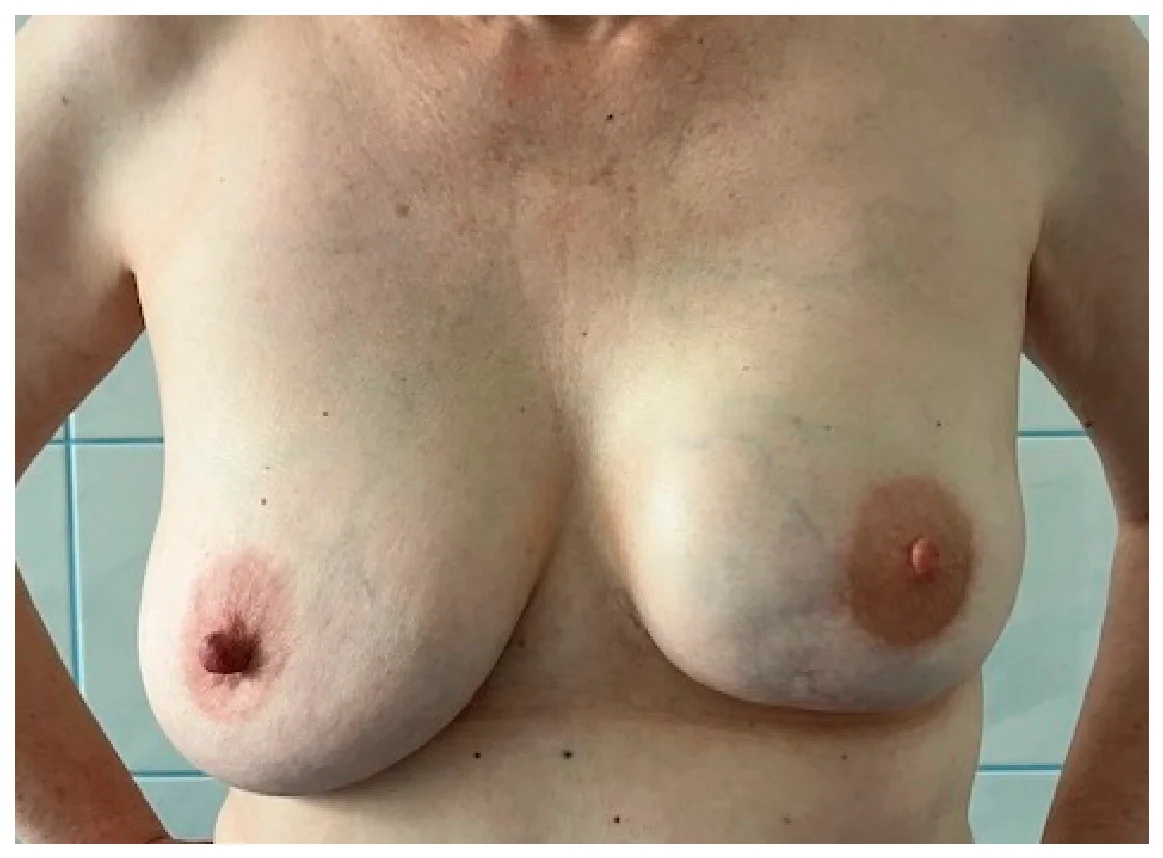
Two years after NSM-LR.

**Figure 25:**
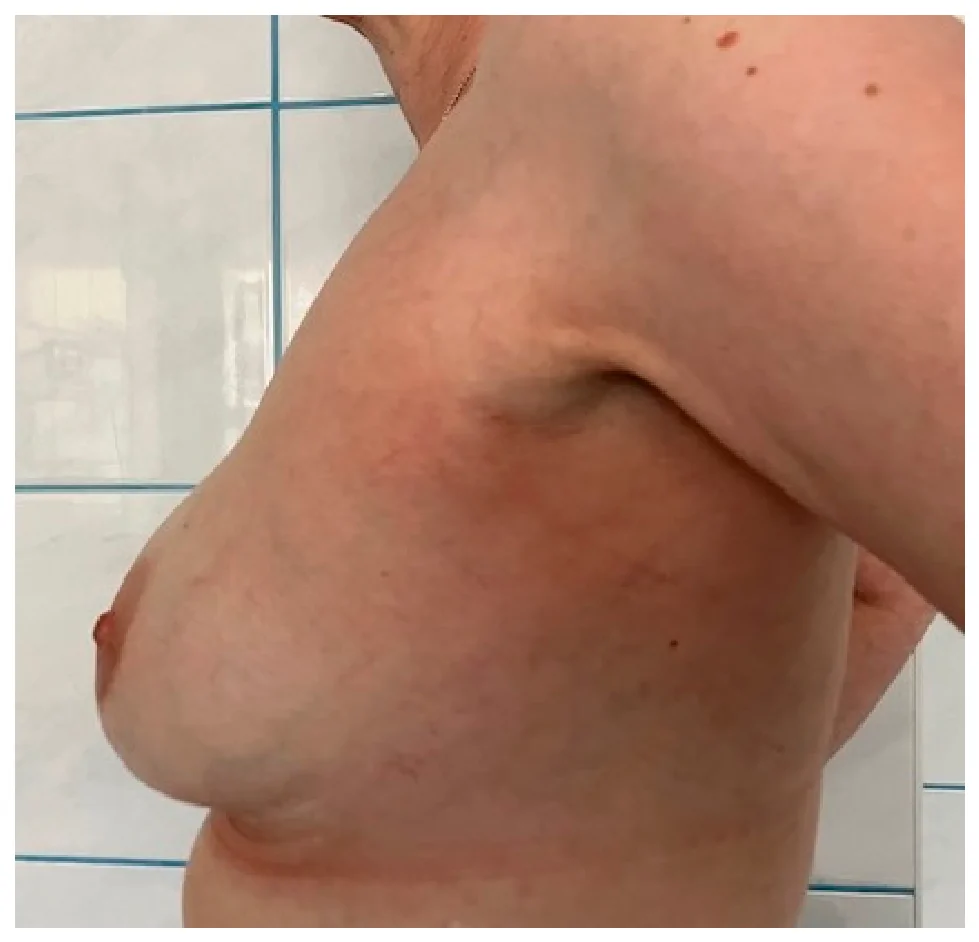
Two years after NSM-LR (left side).

Subsequent photos show different patients months after the NSM-LR procedure, demonstrating a stable result (Figure 22, Figure 23, Figure 24 and Figure 25).

### 2.3. Variable Coding and Selection Procedure

All clinical, pathological, and reconstructive variables were coded according to a dedicated database codifier. The following were analyzed: indication for surgery, surgery duration, implant placement, use of mesh for reconstruction, removed gland and implant volume, complications, implants lost, breast irradiation, recurrence, nipple sensitivity, and patient satisfaction.

Particular attention was paid to complications—categorized into major (requiring surgical intervention) and minor (not requiring intervention)—as well as cases of reconstruction failure (implant loss). Patient satisfaction was assessed according to “BREAST-Q Reconstruction module (postoperative) Version 2.0: Satisfaction with breasts”.

### 2.4. Statistical Analysis

The analysis compares three kinds of operations across two different endpoints. Some endpoints are numbers: age, duration of surgery, removed gland volume, implant volume, and BREAST-Q score. Others are yes/no categories: cancer or prophylaxis, mesh, complication, and implant loss. Numbers and categories call for different tools—hence, two pairs of tests.

Within each pair, the first test answers the question “do the three operations differ at all?”, the second answers “which two of the three?”. The general question comes first, then the detail: this guards against the false findings that inevitably appear if everything is compared with everything at once.

The type of test used to analyze the given variable is presented in Table 1.

### 2.5. Ethics Statement

The study was conducted in accordance with the principles of the Declaration of Helsinki. Owing to the retrospective, non-interventional nature of the analysis, which was based on anonymized routine clinical-care data, individual patient consent was considered not to be required, in accordance with applicable regulations and institutional practice.

## 3. Results

### 3.1. Clinical and Pathological Characteristics of the Patients

A total of 419 post-mastectomy and immediate reconstructions cases were included in the final analysis. They were divided into three analyzed groups based on the surgical technique employed: NSM, NSM-LR, and SRM.

**Figure 26:**
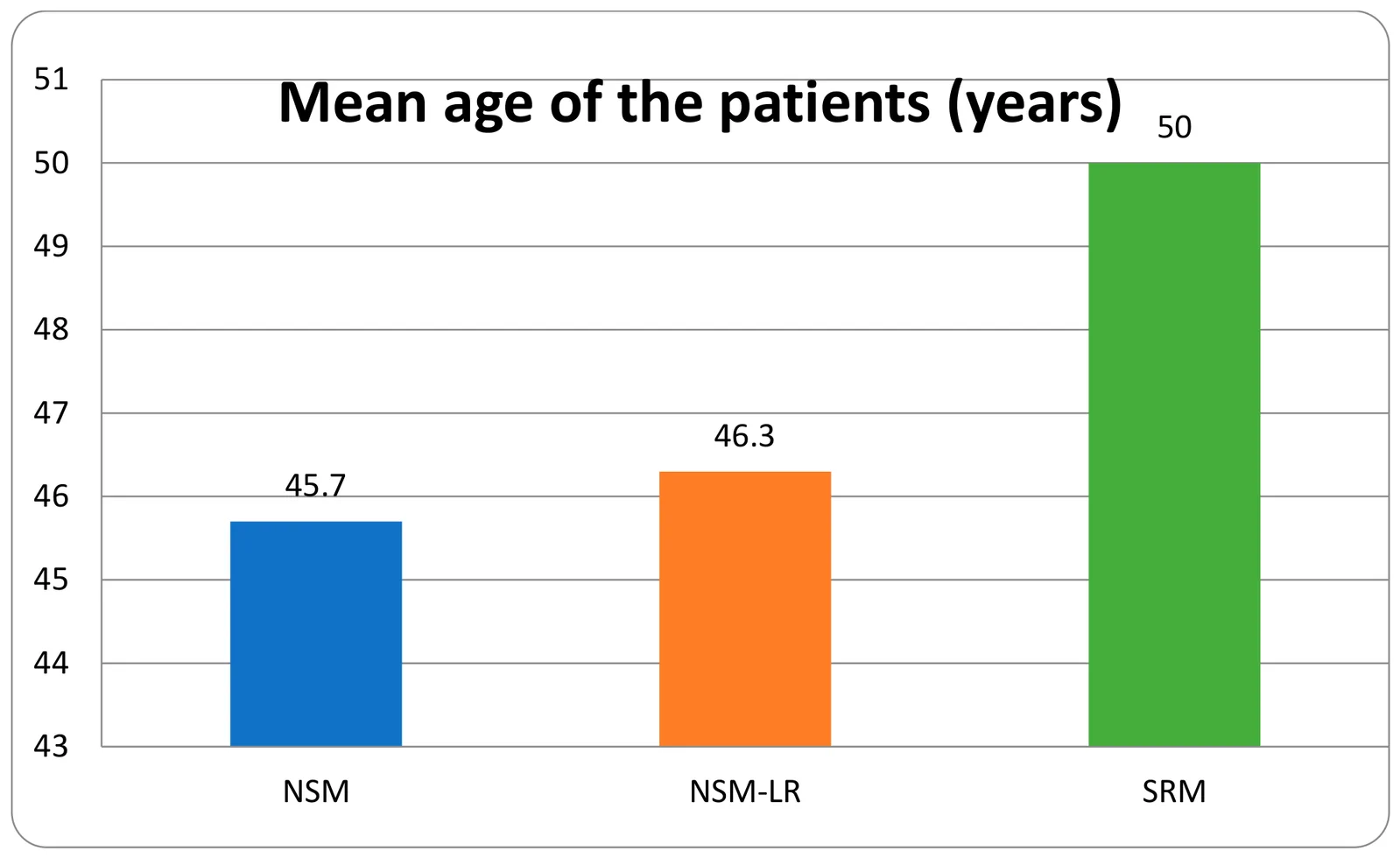
Age distribution in the analyzed groups.

The median age was 44 years in the NSM group (IQR 38–51, *n* = 197), 44.5 years in the NSM-LR group (IQR 38–52, *n* = 80) and 48.5 years in the SRM group (IQR 40–58.8, *n* = 142). The mean age of patients is shown in Figure 26. The difference came from NSM vs. SRM (*p* < 0.001) and NSM-LR vs. SRM (*p* = 0.0218), whereas NSM vs. NSM-LR (*p* = 0.7891) was comparable. The mean age in the SRM group exceeded that in the NSM group by 4.3 years.

**Figure 27:**
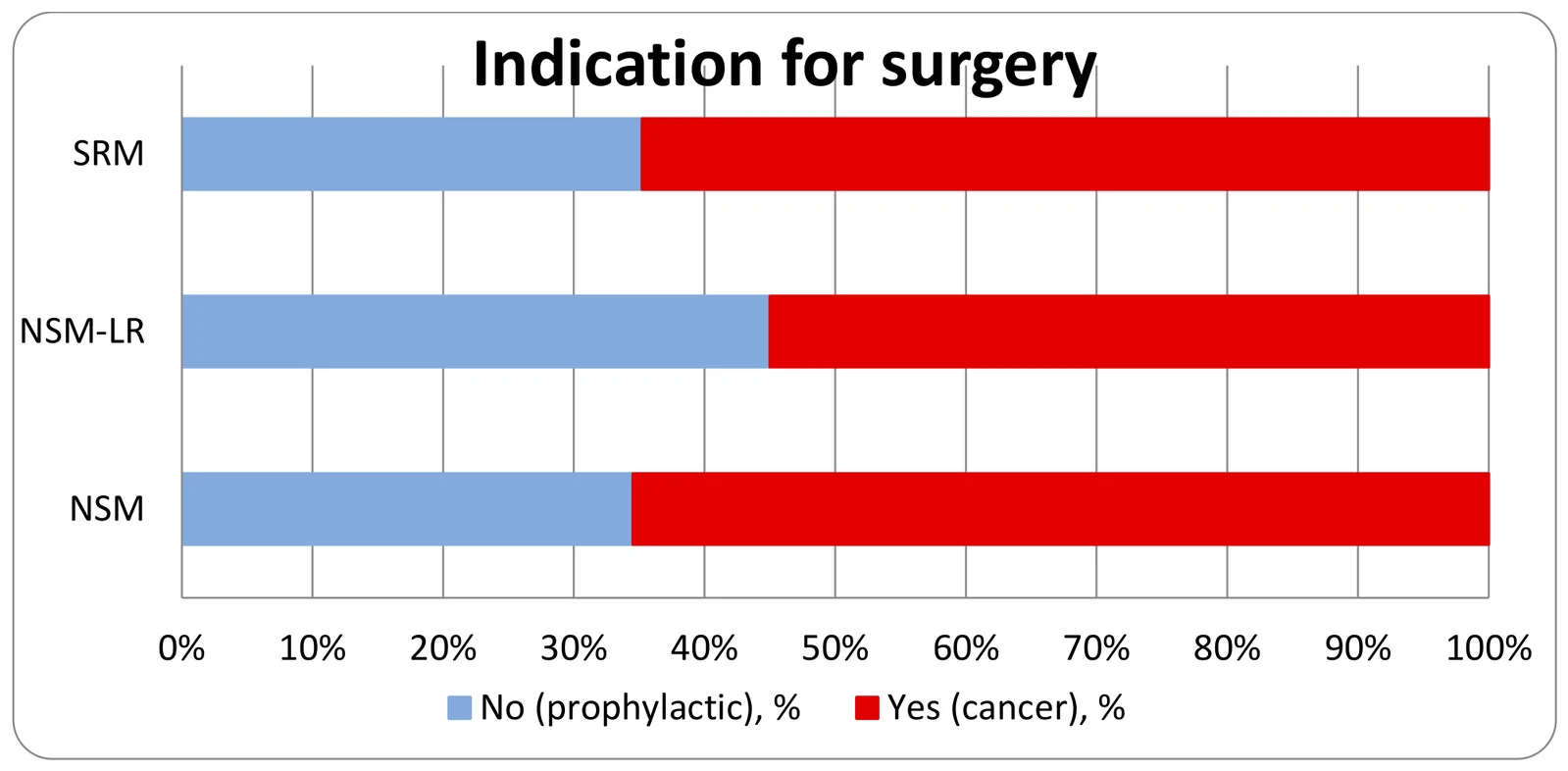
Percentage distribution of surgery indications.

Surgery was performed for cancer in 129/197 cases (65.5%) in the NSM group, 44/80 cases (55.0%) in the NSM-LR group and 92/142 cases (64.8%) in the SRM group. The Pearson chi-square test found no statistically significant difference between the three techniques (χ^2^ = 2.91, df = 2, *p* = 0.2335). The three cohorts were, therefore, comparable in the proportion operated for cancer, which supports the direct comparison of the outcomes. The indication mix is a baseline characteristic of the cohorts rather than a result of surgery (Figure 27).

The detailed clinical and pathological characteristics of patients who underwent surgery for cancer are presented in Table 2.

Among the 265 operations performed for cancer, local recurrence (code 1) occurred in 4/129 (3.1%) after NSM, 4/44 (9.1%) after NSM-LR and 5/92 (5.4%) after SRM (Fisher–Freeman–Halton exact test, *p* = 0.2363), and distant recurrence (code 2) in 20/129 (15.5%) after NSM, 3/44 (6.8%) after NSM-LR and 6/92 (6.5%) after SRM (*p* = 0.0778). Three patients (2 NSM, 1 SRM) had both a local and a distant recurrence; counting these once, overall recurrence affected 39 of the 265 cancer operations (13 local + 29 distant − 3 with both). A higher rate of distant recurrence after NSM than SRM (15.5% vs. 6.5%, *p* = 0.0411) was noted.

A comparison of complications based on prior radiotherapy revealed a significantly higher rate of major complications and implant loss among patients who had undergone chest irradiation, across all analyzed groups (Table 3).

### 3.2. Characteristics of the Surgical Technique

**Figure 28:**
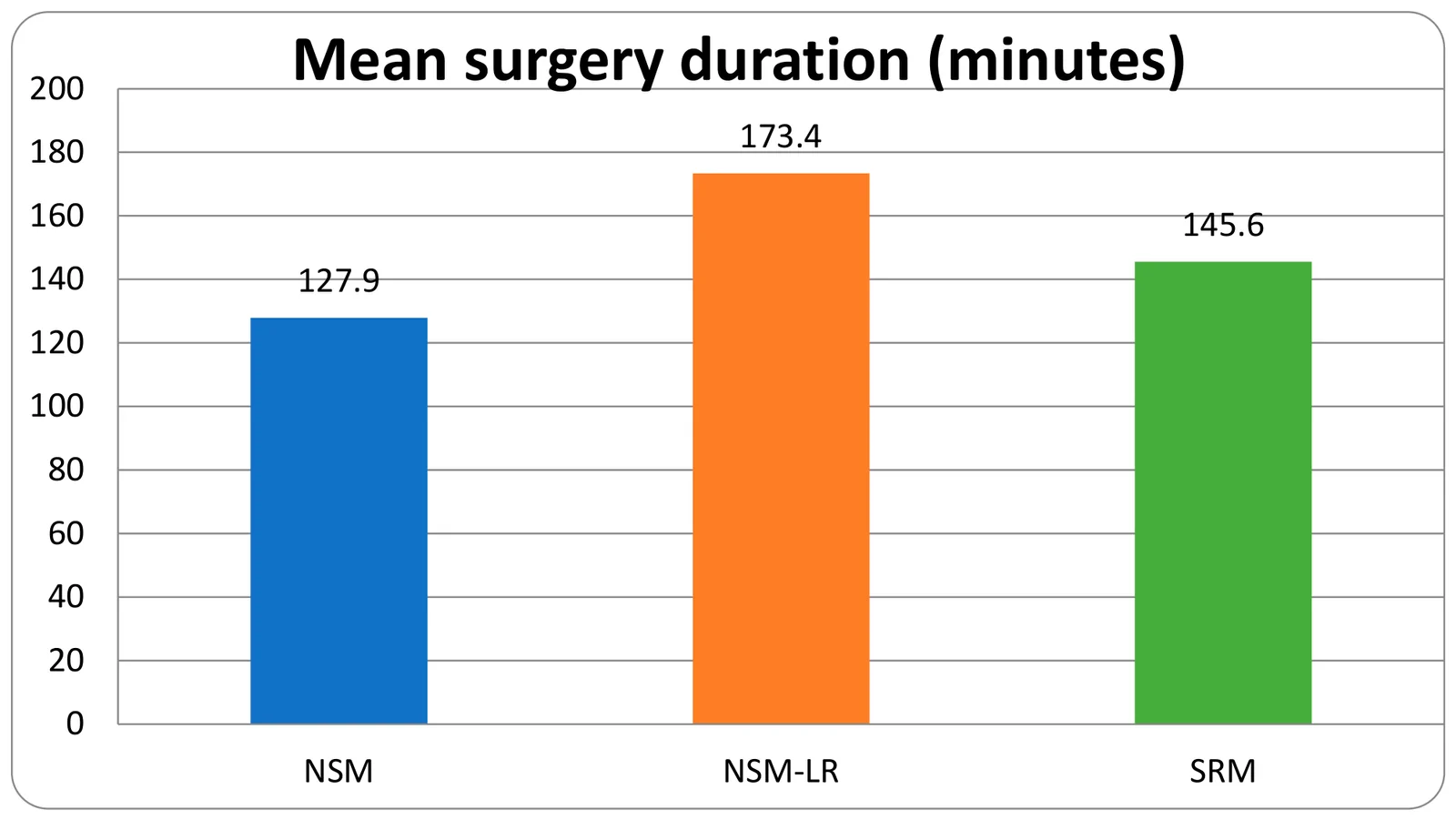
Duration of the surgery.

The Kruskal–Wallis test showed that the surgery durations of the three techniques were not drawn from the same distribution (H = 67.95, df = 2, *p* < 0.001). All three pairwise comparisons were significant: NSM vs. NSM-LR (*p* < 0.001), NSM vs. SRM (*p* < 0.001) and NSM-LR vs. SRM (*p* < 0.001). The mean duration of surgery with NSM-LR exceeded that with NSM by 45.5 min (Figure 28).

**Figure 29:**
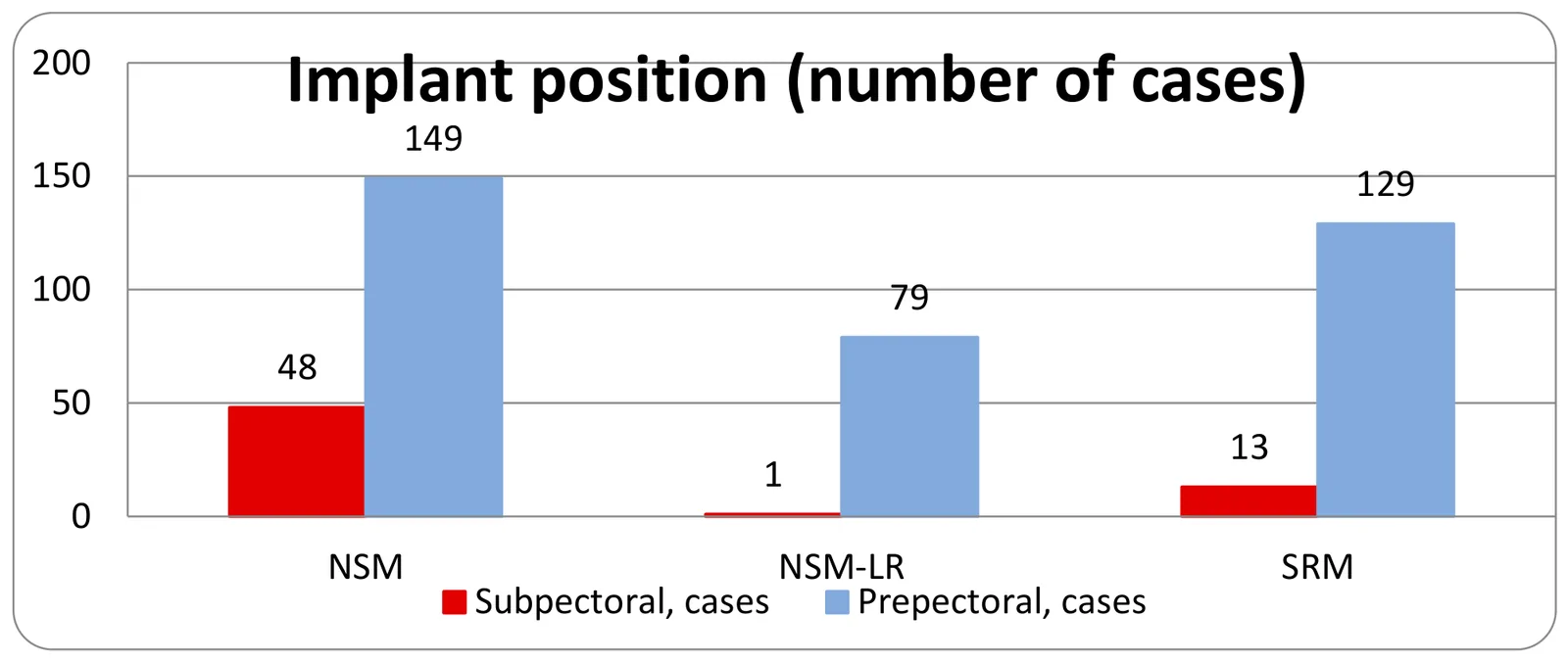
Implant position.

A subpectoral implant position was used in 48/197 cases (24.4%) in the NSM group, 1/80 cases (1.2%) in the NSM-LR group and 13/142 cases (9.2%) in the SRM group. The Pearson chi-square test confirmed that the three techniques differ in this respect (χ^2^ = 29.54, df = 2, *p* < 0.001). All three pairwise comparisons were significant: NSM vs. NSM-LR (*p* < 0.001), NSM vs. SRM (*p* < 0.001) and NSM-LR vs. SRM (*p* = 0.02). The separation between the extremes reached 23.1 percentage points, with NSM showing the highest and NSM-LR the lowest rate of subpectoral placement (Figure 29).

**Figure 30:**
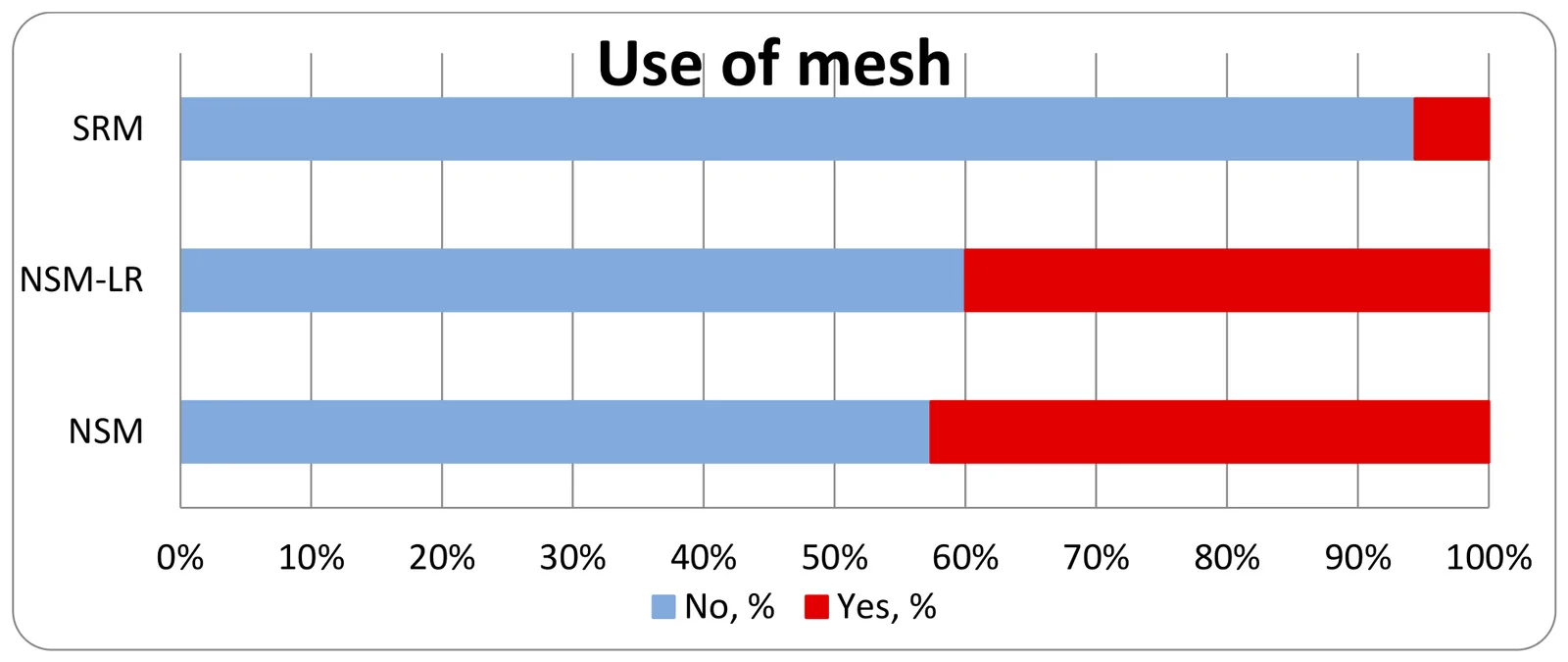
Characteristics of mesh use during reconstruction.

Mesh was used in 84/197 cases (42.6%) in the NSM group, 32/80 cases (40.0%) in the NSM-LR group and 8/142 cases (5.6%) in the SRM group. The Pearson chi-square test confirmed that the three techniques differ in this respect (χ^2^ = 59.37, df = 2, *p* < 0.001). The difference came from NSM vs. SRM (*p* < 0.001) and NSM-LR vs. SRM (*p* < 0.001), whereas NSM vs. NSM-LR (*p* = 0.6865) was comparable. The separation between the extremes reached 37.0 percentage points, with NSM showing the highest and SRM the lowest rate of mesh use (Figure 30).

The median removed gland volume was 250 mL with NSM (IQR 200–308.8, *n* = 174), 320 mL with NSM-LR (IQR 290–400, *n* = 78) and 650 mL with SRM (IQR 500–880, *n* = 128). All three pairwise comparisons were significant: NSM vs. NSM-LR (*p* < 0.001), NSM vs. SRM (*p* < 0.001) and NSM-LR vs. SRM (*p* < 0.001). The mean removed gland volume with SRM exceeded that with NSM by 447.6 mL.

**Figure 31:**
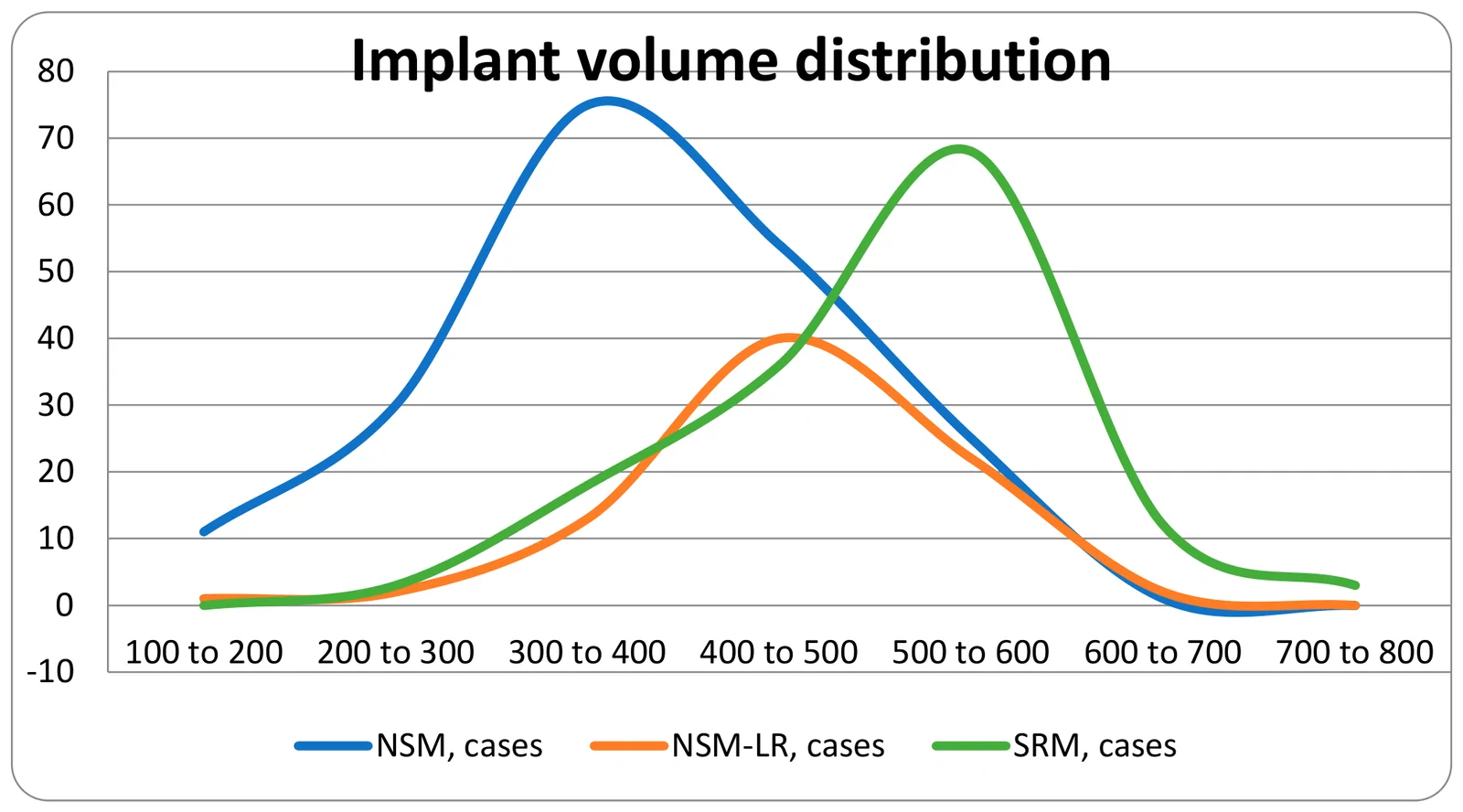
Distribution of implant volume regarding type of surgery technique.

The median implant volume was 375 mL with NSM (IQR 315–450, *n* = 196), 432.5 mL with NSM-LR (IQR 400–500, *n* = 80) and 500 mL with SRM (IQR 448.8–550, *n* = 140). The Kruskal–Wallis test showed that the three techniques were not drawn from the same distribution (H = 114.25, df = 2, *p* < 0.001). All three pairwise comparisons were significant: NSM vs. NSM-LR (*p* < 0.001), NSM vs. SRM (*p* < 0.001) and NSM-LR vs. SRM (*p* < 0.001) (Figure 31).

### 3.3. Complications

**Figure 32:**
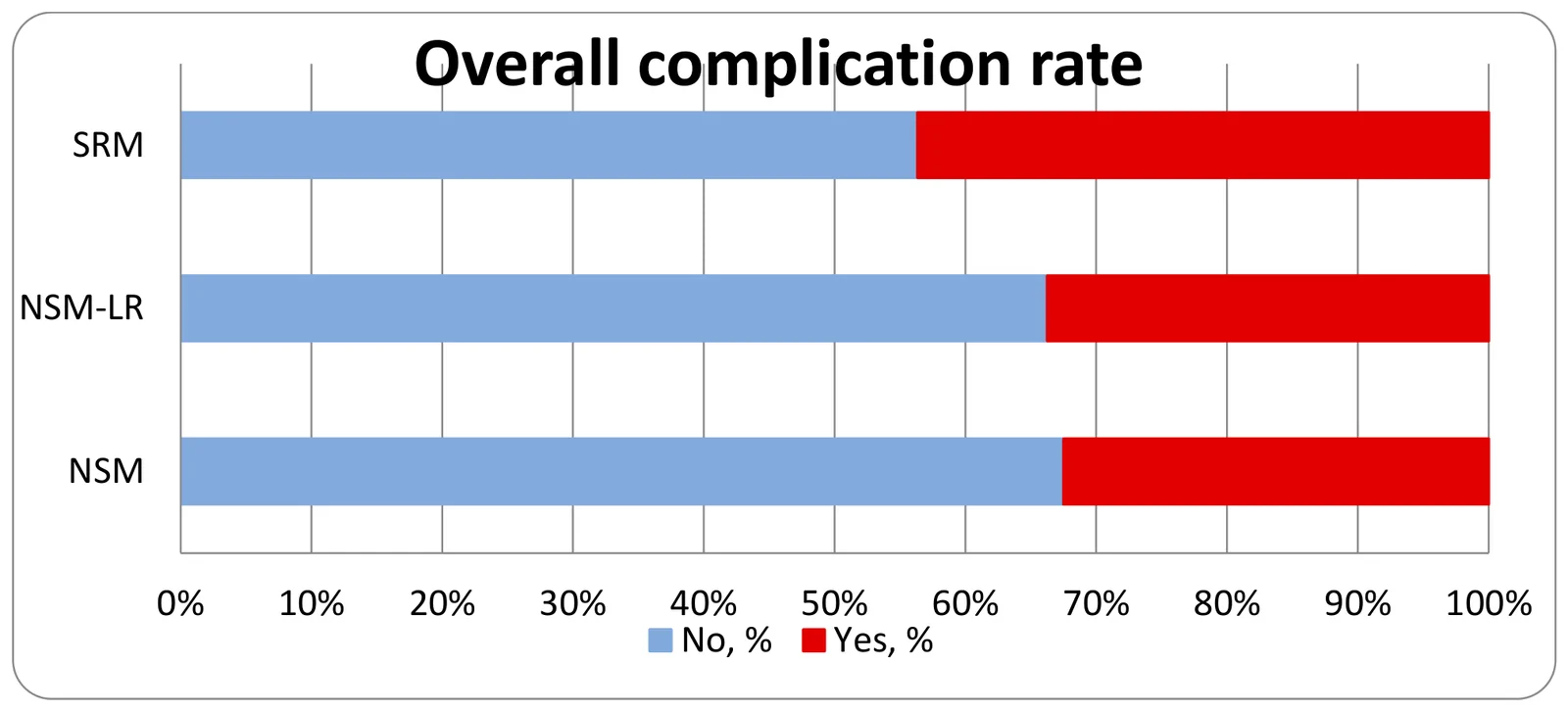
Overall complication rate.

A complication of any severity occurred in 64/197 cases (32.5%) after NSM, 27/80 cases (33.8%) after NSM-LR and 62/142 cases (43.7%) after SRM. The Pearson chi-square test found no statistically significant difference between the three techniques (χ^2^ = 4.77, df = 2, *p* = 0.092). In the pairwise comparisons NSM vs. SRM (*p* = 0.0357) differed nominally, but with the overall test not being significant, this should be read as exploratory rather than confirmatory. The widest separation between any two techniques was 11.2 percentage points (Figure 32).

Minor complications were defined as those that did not require surgical intervention. Healing occurred spontaneously or required the use of specialized dressings. A minor complication occurred in 13/197 cases (6.6%) after NSM, 10/80 cases (12.5%) after NSM-LR and 14/142 cases (9.9%) after SRM. The Pearson chi-square test found no statistically significant difference between the three techniques (χ^2^ = 2.74, df = 2, *p* = 0.2537). None of the three pairwise comparisons reached significance either. The widest separation between any two techniques was 5.9 percentage points. A higher rate of skin necrosis is noted in the case of NSM-LR compared to the other two groups. However, this did not reach statistical significance. The exact number of complications in this group is shown in Table 4.

A major complication occurred in 50/197 cases (25.4%) after NSM, 17/80 cases (21.2%) after NSM-LR and 48/142 cases (33.8%) after SRM. We included in these complications all cases requiring surgical intervention or hospitalization. The Pearson chi-square test found no statistically significant difference between the three techniques (χ^2^ = 4.85, df = 2, *p* = 0.0887). In the pairwise comparisons, NSM-LR vs. SRM (*p* = 0.0485) differed nominally, but with the overall test not being significant, this should be read as exploratory rather than confirmatory. The widest separation between any two techniques was 12.6 percentage points. The exact number of complications in this group is shown in Table 5.

**Figure 33:**
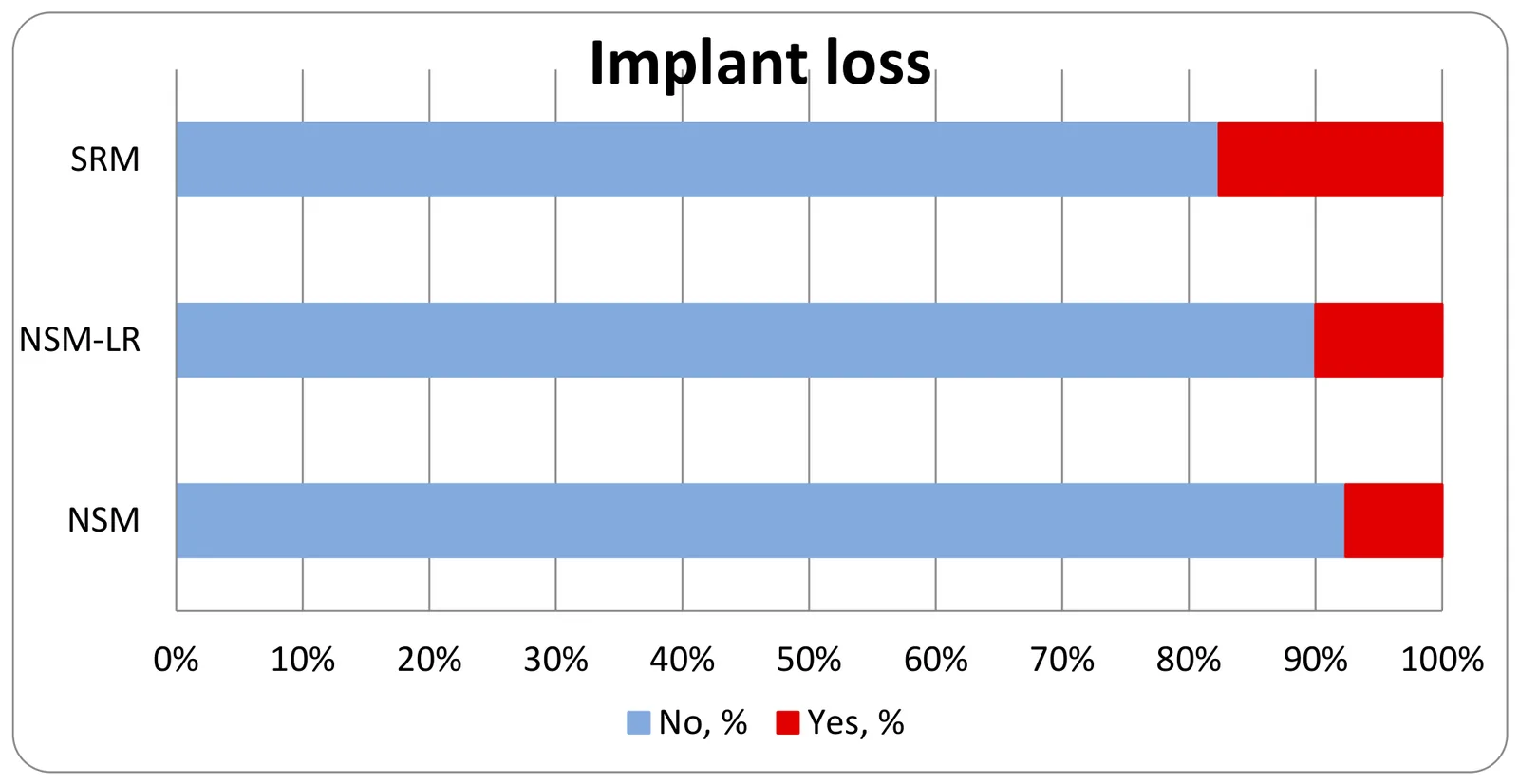
Implant loss rate.

Implant loss occurred in 15/197 cases (7.6%) after NSM, 8/80 cases (10.0%) after NSM-LR and 25/142 cases (17.6%) after SRM. The Pearson chi-square test confirmed that the three techniques differ in this respect (χ^2^ = 8.33, df = 2, *p* = 0.0155). The difference came from NSM vs. SRM (*p* = 0.0049). SRM showed the highest and NSM the lowest rate of implant loss (Figure 33).

### 3.4. Patient Satisfaction

**Figure 34:**
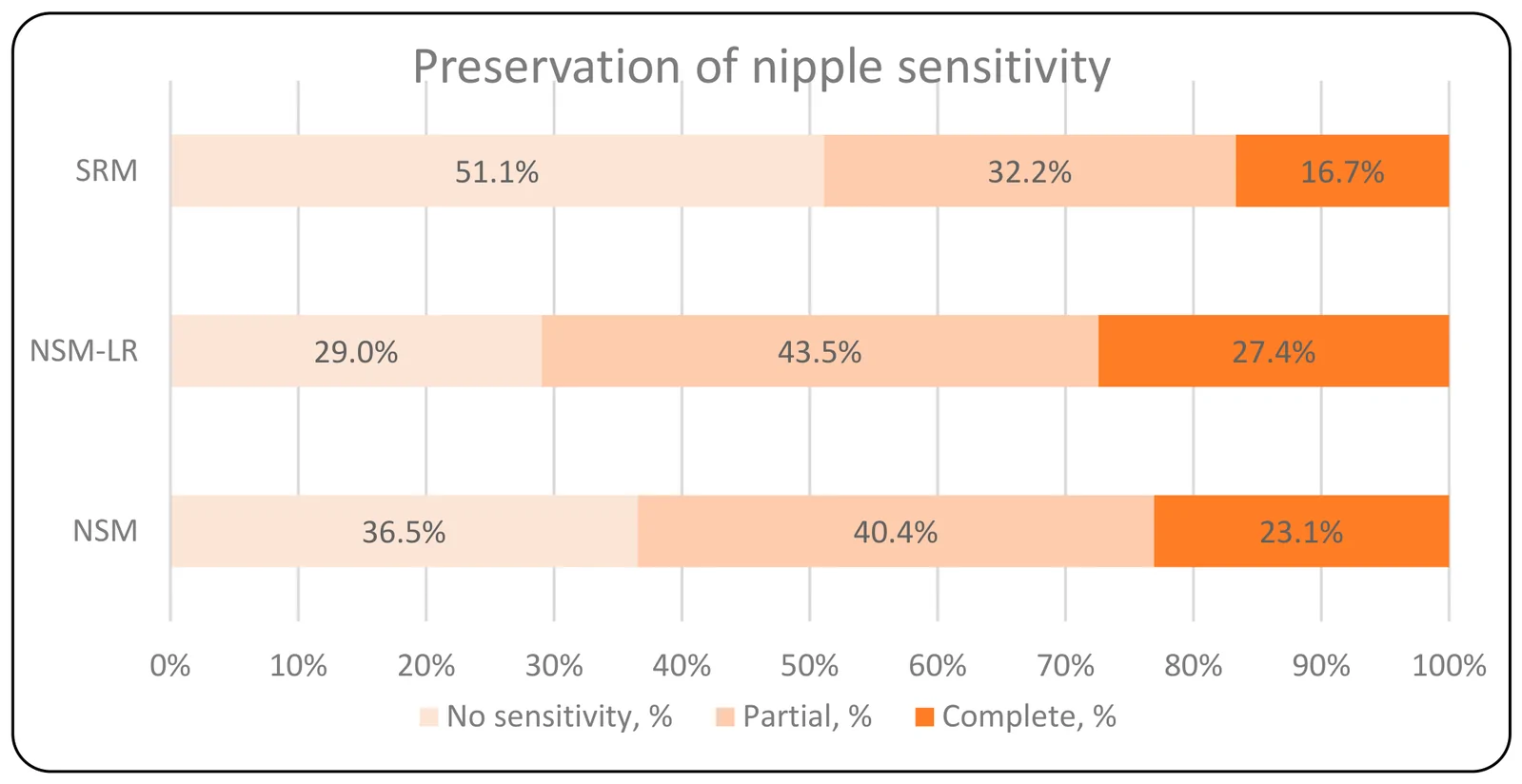
Preservation of nipple sensitivity.

A complete absence of nipple sensitivity was recorded in 57/156 cases (36.5%) after NSM, 18/62 cases (29.0%) after NSM-LR and 46/90 cases (51.1%) after SRM. The Pearson chi-square test found no statistically significant difference between the three techniques (χ^2^ = 8.70, df = 4, *p* = 0.0691) (Figure 34).

The median BREAST-Q score was 78 points after NSM (IQR 64.2–98, *n* = 162), 82 points after NSM-LR (IQR 69–100, *n* = 62) and 75 points after SRM (IQR 64–98, *n* = 102). The Kruskal–Wallis test found no statistically significant difference between the three techniques (H = 2.02, df = 2, *p* = 0.3637). None of the three pairwise comparisons reached significance either (Table 6).

## 4. Discussion

### 4.1. Discussion of Results

We retrospectively analyzed all patients who underwent surgery at our hospital between 2016 and 2025 using a technique involving immediate reconstruction. No inclusion criteria other than this single criterion were applied. Due to insufficient data, only 17 out of 436 cases were excluded from the analysis, representing a data loss of just 3.9%. This constitutes one of the strengths of our analysis. Consequently, we estimate that within our center’s patient population, the target group for the method we describe comprises 19% of all female patients requiring subcutaneous mastectomy with immediate reconstruction. When comparing the analyzed groups of patients, we observed a significantly higher median age in the group undergoing surgery involving classic skin reduction. In our opinion, this is attributable to natural bodily changes; older patients are more likely to have ptotic breasts that require the removal of excess skin during surgery. This is confirmed by a publication from the University of Kentucky, which analyzed risk factors for ptotic breasts [17].

The high proportion of prophylactic surgeries across the entire cohort reflects the specific nature of our breast center. We perform most of these as bilateral procedures in patients diagnosed with breast cancer who are at high familial risk of developing cancer in the contralateral breast (e.g., carriers of BRCA gene mutations). The groups analyzed did not differ in terms of the ratio of prophylactic surgeries to surgeries for diagnosed cancer. In our view, this approach allowed us to avoid the risk that prior oncological treatment might have influenced the analysis results. Based on the available literature, we know that perioperative systemic therapy does not affect the rate of postoperative complications [18]. However, radiotherapy—whether administered prior to mastectomy (e.g., following prior breast-conserving therapy) or after subcutaneous mastectomy—significantly impacts surgical outcomes [19,20]. This is confirmed by our own findings, which showed that the incidence of major complications was nearly twice as high and the rate of implant loss more than three times higher in the group of patients who had undergone chest-wall irradiation. Since the distribution of radiotherapy was similar across all three comparison groups, we can assume that it did not influence the comparative analysis of the surgical technique itself. In cases of surgery performed for malignancy, radical resection (R0) was achieved in all patients. Local recurrence rates were similar, suggesting that all the surgical techniques analyzed ensure the same level of “oncological clearance.” However, a notably higher rate of distant recurrence was observed in the NSM group. Despite a meticulous analysis of the collected data, we were unable to identify the cause of this finding. The distribution of disease stage, as well as histological and molecular subtypes, was comparable across all three groups. This result is also inconsistent with the literature, which indicates no significant differences in the incidence of distant metastases based on the surgical technique used [21,22]. During the analysis of data regarding the technical aspects of the compared procedures, we identified significant differences in implant positioning relative to the pectoralis major muscle. Published data indicate a lower complication rate with prepectoral implant placement [23,24]. Our results demonstrate the same pattern. The highest rate (24.4%) of submuscular implant placement was observed in the NSM group, which also showed the highest incidence (9.14%) of capsular contracture requiring surgical revision. Conversely, the lowest percentage (1.2%) of submuscular implant placement was found in the NSM-LR group, which also recorded the lowest rate (2.5%) of severe capsular contracture. Implant migration also occurred more frequently in the group with the highest number of subpectoral implant positions. Given these findings, it must be concluded that the differences in these two types of complications stem from the implant’s position relative to the pectoralis major muscle rather than the surgical technique employed. Differences in the use of a mesh to additionally cover or stabilize the implant stemmed from variations in surgical techniques. During SRM, the implant—or its lower part—is covered by a buried, de-epithelialized skin flap from the lower quadrants of the breast; consequently, this flap “replaces” the mesh, rendering its use unnecessary [12]. At our center, we do not use biological meshes, employing only synthetic ones. According to the literature, biological meshes are associated with a higher incidence of seromas and infectious complications, whereas synthetic meshes do not increase the rate of such complications [25,26]. In our analysis, we observed a higher infection rate (6.34%) in the SRM group—where mesh usage was less frequent—compared to the NSM (1.52%) and NSM-LR (3.75%) groups, where mesh utilization was higher. Although this difference was not statistically significant, it confirms that the use of mesh in groups not involving skin excision did not adversely affect outcomes. The mean volume of excised glandular tissue and the mean volume of the implant used for reconstruction confirm that the patients selected for the surgical technique described here had larger breasts and required larger implants; effectively, they constituted a group positioned between patients undergoing surgery without reduction and those requiring the excision of a large excess of skin. Ideally, it would also be useful to compare the degree of ptosis and pre-operative breast volume; however, we did not collect such data. These aspects could be the subject of future research aimed at establishing an objective method for identifying patients who require a reduction in the envelope but can avoid skin excision. Our earlier observations of a higher rate of complications in the group undergoing excision of excess skin were confirmed, though the findings did not reach statistical significance. The results of this analysis indicate that the primary complication encountered at our center is ischemia or necrosis of the skin flaps. We also included postoperative wound healing issues—which stem directly from poor tissue perfusion—in this category of complications [27,28]. This is evident in the analysis of both minor and major complications. In the NSM-LR group, we observed a higher incidence of minor complications classified as skin necrosis or skin ischemia, confirming our clinical impression of more frequent wound-healing issues in the periareolar region. The introduction of minimally invasive procedures did not alter this trend. Within the NSM-LR group, 15 procedures were performed using an endoscopic technique; in three cases (20%), impaired wound healing was observed around the NAC. The insufficient number of procedures performed using the endoscopic technique precludes drawing statistically significant conclusions, but it appears that the technique has no impact on the incidence of wound dehiscence around the NAC. Previous studies on incision placement have shown that the periareolar incision is a primary factor contributing to postoperative wound healing complications [11,29]. Since we performed only de-epithelialization rather than a full-thickness skin incision, most of these complications resolved spontaneously and did not lead to reconstruction failure, resulting only in a wider scar around the nipple–areola complex. The author recommends using interrupted sutures around the NAC. Observations suggest that a continuous intradermal suture may be associated with microcirculatory disturbances along the suture line, caused by increased tension from the underlying implant [30]. This can result in impaired wound healing around the nipple–areola complex (NAC). Since this is merely a subjective assessment not backed by a detailed analysis, it could be the subject of future analysis.

The reason for introducing a surgical technique at our center nearly a decade ago—one that allows for the reduction in the skin envelope without excising excess skin—was the observation of significant necrosis within the postoperative wound. This occurred primarily near the vertical incision line used in the “Wise pattern” technique. The results of this analysis confirm that eliminating this incision, combined with the decision not to excise excess skin, led to a reduction in major complications among patients requiring a reduction in the implant pocket. A higher incidence of necrotic complications requiring reoperation and a higher rate of implant loss were observed in the SRM group. In contrast, the NSM-LR group achieved results comparable to those of the NSM group (which involved no skin reduction). Operations involving the excision of skin and subcutaneous tissue damage the vessels that supply blood to the skin flaps remaining after the breast gland is removed. In our opinion, avoiding a full-thickness skin incision preserved some of these vessels, resulting in fewer serious complications following reduction surgery. Further research focusing on the precise measurement and comparison of blood supply to the remaining tissues could provide accurate and measurable (objective) evidence to confirm this hypothesis. Immediate breast reconstruction has become a widely used technique for patients requiring mastectomy, owing to the benefits it offers in preserving body image integrity [5,6]. The aesthetic quality of the reconstructed breast has a profound impact on psychological, social, and sexual quality of life. In 2009, a comprehensive questionnaire for assessing the quality of life of patients following breast surgery—BREAST-Q—was made available [31]. To determine whether the use of the new surgical technique affected long-term aesthetic outcomes, we asked our patients to complete the BREAST-Q questionnaire regarding postoperative satisfaction with the reconstructed breast. The results obtained were similar across all three groups. High BREAST-Q scores confirmed that the proposed technique ensures high-quality aesthetic outcomes. The nipple is a key erogenous zone, and the preservation of sensation within it impacts quality of life [32,33]. We asked our patients about the level of preserved nipple sensation. As expected, the level of preserved nipple sensation was comparable between the two groups that did not undergo skin excision and was higher than in the SRM group. This is because, during surgery using the “Wise pattern” technique, the nipple–areola complex is repositioned to the center of the reconstructed breast either on a skin pedicle or as a free skin graft. Preserving the pedicle can prevent damage to some sensory nerve branches, whereas the use of a skin graft completely severs them, resulting in a denervated nipple.

### 4.2. Study Limitations, Strong Points and Clinical Implications

Our study has limitations that should be taken into account when interpreting the results. First, it is a retrospective, single-center analysis, subject to the inherent risk of selection bias and data incompleteness. Second, the assessment of the aesthetic outcome and nipple sensation was subjective, and the BREAST-Q questionnaire was administered only once—without a baseline assessment or breakdown into individual domains. Third, a high rate of distant recurrence in the NSM group suggests the presence of an unknown variable influencing oncological outcomes. Fourth, differences in implant placement relative to the pectoral muscle can influence the overall complication rate and satisfaction levels.

A definite strength of this analysis is the minimal loss of data; only 3.9% of cases from the original database were excluded due to incomplete data. Furthermore, the size of the entire cohort is among the largest reported in single-center analyses. Historical groups were not involved in the comparison, ensuring that the team’s experience and equipment capabilities could not influence the analysis results.

Despite the limitations, our observations have practical clinical implications. They demonstrate that in some patients, instead of using larger implants to fill the skin envelope, the envelope can be reduced without excising excess skin. They also demonstrate that this procedure does not affect oncological outcomes and leads to a reduction in serious complications.

Further studies are needed to confirm the results of this study—specifically, multicenter trials that eliminate single-center bias. Future research comparing skin flap vascularity (e.g., using indocyanine green) between the technique involving excess skin excision and the de-epithelialization-only method would also help demonstrate that improved blood supply is the reason for the lower rate of necrotic complications.

## 5. Conclusions

Reducing the skin envelope without excising excess skin during nipple-sparing mastectomy with immediate implant-based reconstruction may be associated with fewer major complications and implant losses.

NSM-LR may be a reasonable option for a selected group of patients requiring skin-reducing mastectomy.

## Figures and Tables

**Table 1:** Statistical tests used for the analyzed variables.

What Is Compared	Type of Data	Test
Age, duration of surgery, gland volume, implant volume, BREAST-Q	Numbers	Kruskal–Wallis (3 groups) → Mann–Whitney (pairs)
Cancer, implant position, mesh, complications, implant loss, nipple sensitivity, RT	Yes/no categories	Pearson’s chi-square
Recurrence—local, distant, overall	Categories, but very few events	Fisher–Freeman–Halton exact test

**Table 2:** Clinical and pathological characteristics of patients who underwent surgery as a cancer treatment.

Variable	NSM	NSM-LR	SRM	*p*
Age (years)				
Mean ± SD	45.7 ± 10.4	46.3 ± 10.4	50.3 ± 10.0	*p* = 0.0025
Median (range)	44	44.5	48.5	
Indication—*n* (%)				
Breast cancer	129 (65.5%)	44 (55%)	92 (64.8%)	*p* = 0.2335
Prophylaxis	68 (34.5%)	36 (45%)	50 (35.2%)	
Histological type—*n* (%)				
Invasive carcinoma NST	88 (68.2%)	34 (77.3%)	55 (59.8%)	
Invasive lobular carcinoma	22 (17.1%)	6 (13.6%)	15 (16.3%)	NA
DCIS	14 (10.8%)	3 (6.8%)	15 (16.3%)	
Other (mucinous, metaplastic, medullary)	4 (3.1%)	1 (2.3%)	7 (7.6%)	
Molecular subtype—*n* (%)				
Luminal A	42 (35.9%)	10 (24.3%)	30 (38.5%)	
Luminal B HER2−	23 (19.7%)	9 (22%)	11 (14.1%)	
Luminal B HER2+	11 (9.4%)	5 (12.2%)	12 (15.4%)	NA
Non-luminal HER2+	8 (6.8%)	2 (4.9%)	4 (5.1%)	
TNBC	33 (28.2%)	15 (36.6%)	21 (26.9%)	
Resection margins—*n* (%)				
Positive	0 (0%)	0 (0%)	0 (0%)	
Negative	129 (100%)	44 (100%)	92 (100%)	
Radiotherapy—*n* (%)				
Yes	44 (22.3%)	16 (20%)	32 (22.5%)	*p* = 0.8945
Recurrence				
Local recurrence	4 (3.1%)	4 (9.1%)	5 (5.4%)	*p* = 0.2363
Distant recurrence	20 (15.5%)	3 (6.8%)	6 (6.5%)	*p* = 0.0411

NA: Not analyzed.

**Table 3:** Complication vs. radiotherapy.

Complication	RTH Given	RTH Not Given	Difference	*p*-Value	Result
Any complication	41/92 (44.6%)	112/327 (34.3%)	10.3 percentage points more with RTH	0.0695	Not significant
Minor complication	5/92 (5.4%)	32/327 (9.8%)	4.4 percentage points fewer with RTH	0.1938	Not significant
Major complication	37/92 (40.2%)	78/327 (23.9%)	16.4 percentage points more with RTH	0.0019	Significant
Implant loss	24/92 (26.1%)	24/327 (7.3%)	18.7 percentage points more with RTH	6.11 × 10^−7^	Significant

**Table 4:** Minor complications.

Complication Type (No. Cases)	NSM	NSM-LR	SRM	Total
Skin/flap necrosis/wound dehiscence	8	9	10	27
Hematoma	0	0	1	1
Infection	1	0	0	1
Capsular contracture	3	0	1	4
Implant migration	1	1	2	4

**Table 5:** Major complications.

Complication Type (No. Cases)	NSM	NSM-LR	SRM	Total
Skin/flap necrosis/wound dehiscence	15	9	28	52
Hematoma	7	3	7	17
Infection	3	3	9	15
Capsular contracture	18	2	3	23
Implant migration	5	0	0	5
Implant rupture	3	0	3	6
Chronic pain syndrome	1	0	0	1

**Table 6:** BREAST Q score.

Group	No. Cases	Mean	SD	Median
NSM	162	77.4	18.7	78
NSM-LR	62	80.2	18.4	82
SRM	102	75.5	20.1	75
All groups	326	77.4	19.1	78

## Data Availability

The data supporting the findings of the study are available from the corresponding author upon reasonable request.

## Abbreviations

The following abbreviations are used in this manuscript:

NSM	Nipple-Sparing Mastectomy
NSM-LR	Nipple-Sparing Mastectomy with Limited Reduction
SRM	Skin-Reducing Mastectomy
NST	Non-Special Type
DCIS	Ductal Carcinoma In Situ
NAC	Nipple–areola Complex
LVC	Lower Vertical Curvature
RT	Radiotherapy
